# Photovoltage enhancement of M-series acceptor-based polymer solar cells and minimodules through the modulation of charge-transfer states

**DOI:** 10.1093/nsr/nwaf089

**Published:** 2025-03-07

**Authors:** Kaichen Xing, Dongdong Cai, Di Wang, Jin-Yun Wang, Changquan Tang, Yunlong Ma, Qingdong Zheng

**Affiliations:** State Key Laboratory of Structure Chemistry, Fujian Institute of Research on the Structure of Matter, Chinese Academy of Sciences, Fuzhou 350002, China; College of Chemistry, Fuzhou University, Fuzhou 350116, China; Fujian College, University of Chinese Academy of Sciences, Fuzhou 350002, China; State Key Laboratory of Structure Chemistry, Fujian Institute of Research on the Structure of Matter, Chinese Academy of Sciences, Fuzhou 350002, China; State Key Laboratory of Structure Chemistry, Fujian Institute of Research on the Structure of Matter, Chinese Academy of Sciences, Fuzhou 350002, China; State Key Laboratory of Structure Chemistry, Fujian Institute of Research on the Structure of Matter, Chinese Academy of Sciences, Fuzhou 350002, China; State Key Laboratory of Structure Chemistry, Fujian Institute of Research on the Structure of Matter, Chinese Academy of Sciences, Fuzhou 350002, China; Fujian College, University of Chinese Academy of Sciences, Fuzhou 350002, China; State Key Laboratory of Structure Chemistry, Fujian Institute of Research on the Structure of Matter, Chinese Academy of Sciences, Fuzhou 350002, China; Fujian College, University of Chinese Academy of Sciences, Fuzhou 350002, China; State Key Laboratory of Coordination Chemistry, College of Engineering and Applied Sciences, Nanjing University, Nanjing 210023, China

**Keywords:** organic solar cell, photovoltaic module, non-fullerene acceptor, charge-transfer state, side-chain fluorination, energy loss

## Abstract

Side-chain fluorination can enhance the backbone organization and carrier mobility of non-fullerene acceptors (NFAs) but it often reduces their photovoltage due to the resulting deeper-lying lowest unoccupied molecular orbital (LUMO) levels. Herein, we present a strategy to regulate the LUMO levels of two NFAs, MC9F5 and MC7F5, by repositioning the highly electronegative –C_2_F_5_ moieties on the side chains. This approach mitigates the impact of fluorination on the energy levels, thereby improving the photovoltage and overall device performance. By incorporating 10,10,11,11,11-pentafluoro-2-(8,8,9,9,9-pentafluorononyl)undecyl side chains, the –C_2_F_5_ moieties are positioned away from the conjugated backbone of MC9F5, resulting in an elevated LUMO level compared with MC7F5, which features 8,8,9,9,9-pentafluoro-2-(6,6,7,7,7-pentafluoroheptyl)nonyl side chains. This modification reduces both the charge generation and the non-radiative energy losses in the MC9F5-based devices. The MC9F5-based small-area and minimodule devices achieve efficiencies of 18.02% and 15.66%, respectively, which are among the highest values reported for acceptor–donor–acceptor-type NFAs. This study highlights a valuable fluorination strategy for achieving high-performance NFAs.

## Teaser

By strategically relocating the electronegative -C_2_F_5_ groups on side-chains of M-series acceptors, we developed an effective approach to precisely modulate their energy levels thereby enhancing photovoltage and overall device performance.

## INTRODUCTION

Organic solar cells (OSCs) represent a promising complementary photovoltaic technology to the currently dominant silicon-based solar cells, offering distinct advantages such as flexibility, light weight, solution processability and semitransparency [1–7]. These characteristics make OSCs particularly well suited for applications such as building-integrated photovoltaics, automotive components and various portable power sources for both indoor and outdoor use [8–12]. Recently, the power conversion efficiencies (PCEs) of OSCs have significantly improved due to the emergence of non-fullerene acceptors (NFAs), which feature long-wavelength absorption capabilities and minimized energy losses when blended with wide-bandgap copolymers [13–18]. The adoption of NFAs has markedly enhanced the photovoltaic performance of OSCs by increasing both photocurrent density and voltage output [13–17]. In 2019, a benchmark fused-ring NFA, Y6, with an acceptor–donor–acceptor–donor–acceptor (ADA'DA) configuration, was reported to have achieved an outstanding efficiency of 15.7% [18]. Since then, extensive efforts have been made by many research groups to chemically modify Y6, leading to the realization of several high-performance ADA'DA-type NFAs, such as L8-BO, N3 and BTP-eC9 [19–21]. To date, the majority of OSCs that demonstrate PCEs of >18% have predominantly employed Y6-series acceptors, whereas high-performance OSCs that utilize ADA-type NFAs remain comparatively scarce. Therefore, it is essential to develop new molecular design strategies to expand the range of high-performance NFA materials.

In 2020, our group reported a new class of acceptors known as M-series acceptors, which are based on an ADA-type architecture and synthesized by substituting all sp³-hybridized bridging carbon atoms in the ladder-type donor core with sp²-hybridized bridging nitrogen atoms [22–25]. The M-series acceptors demonstrate reduced synthetic complexity and exhibit a superior figure of merit compared with several representative NFAs, such as IT-4F and Y6 [26]. Furthermore, these linear-shaped ADA-type NFAs show extended light absorption and an ordered 3D network of molecular stacking, which is facilitated by enhanced intermolecular π–π interactions [27]. Initially, the M-series acceptors achieved a best PCE of 15.2%, which was subsequently improved to 17% through molecular structure optimization and morphology control [22,27]. More recently, we introduced partially fluorinated and branched side chains to enhance the backbone organization of the M-series acceptor (MC7F3) [28]. This modification facilitated the formation of a 3D network packing structure, thereby enhancing carrier transport and boosting the device efficiency to 17.61%. However, fluorinated side chains, while enhancing short-circuit current density (*J*_sc_) and fill factor (FF), induce a downshift in the lowest unoccupied molecular orbital (LUMO) level, consequently lowering the charge-transfer (CT) state and open-circuit voltage (*V*_oc_) of the devices. It remains challenging to leverage the positive effects induced by side-chain fluorination while mitigating their adverse effects.

In contrast to non-excitonic solar cells (e.g. inorganic semiconductor cells), in which excitons with low binding energies can be dissociated by thermal energy, excitonic OSCs involve the CT state due to the low dielectric constant of organic materials [29–32]. It has been disclosed that the CT state energy is approximately equal to the energy difference between the LUMO of the acceptor material and the highest occupied molecular orbital (HOMO) of the donor material [33]. The presence of a CT state results in an additional energy-loss channel, Δ*E*_CT_, and a significant non-radiative energy loss, Δ*E*_non-rad_. The Δ*E*_CT_ represents the energy loss associated with charge generation, linked to the essential driving force for exciton dissociation in OSCs. It can be calculated by using the equation of Δ*E*_CT_ = *E*_g_ − *E*_CT_, where *E*_g_ is the optical bandgap of the donor/acceptor materials and *E*_CT_ is the energy of the CT state [33]. Thus, increasing *E*_CT_ by minimizing the energetic offset between the donor and acceptor materials can effectively reduce Δ*E*_CT_ [33,34]. Importantly, the reduction in Δ*E*_CT_ can enhance the hybridization between the highly luminescent localized excitation (LE) state and the CT state [35,36]. Through an intensity borrowing mechanism, this can increase the luminescence of the CT state, thus decreasing Δ*E*_non-rad_ [36]. However, the reduction of Δ*E*_CT_ would also increase the risk of insufficient exciton dissociation and charge generation, leading to a decrease in external quantum efficiency (EQE) [37–39]. Thus, the precise modulation of *E*_CT_ in OSCs is fundamentally important for energy-loss reduction and device performance optimization.

**Figure 1.**
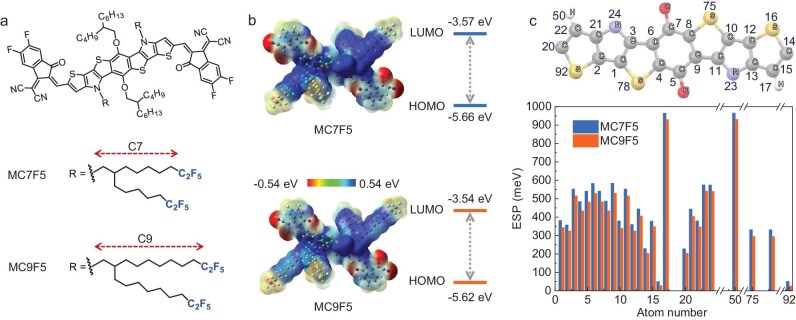
(a) Chemical structures of MC7F5 and MC9F5. (b) Theoretical calculations of the ESP distributions and energy levels of MC7F5 and MC9F5. (c) Labeling numbers for each atom in the fused-ring skeleton (top) and average ESP of each atom on fused-ring skeleton calculated on the B3LYP/6–31G** level (bottom).

Herein, we strategically designed and synthesized two partially fluorinated and branched side chains, namely 8,8,9,9,9-pentafluoro-2-(6,6,7,7,7-pentafluoroheptyl)nonyl and 10,10,11,11,11-pentafluoro-2-(8,8,9,9,9-pentafluorononyl)undecyl groups, which were incorporated into the M-series acceptor framework, resulting in two new NFAs (MC7F5 and MC9F5 in Fig. 1). Moving the strongly electronegative –C_2_F_5_ terminal groups away from the conjugated skeleton leads to a lower surface electrostatic potential and an elevated LUMO energy level for MC9F5 in comparison with those for MC7F5. This chemical modification elevates the CT state and reduces the gap between *E*_g_ and *E*_CT_, effectively lowering both Δ*E*_CT_ and Δ*E*_non-rad_, which contributes to an increased *V*_oc_ in the MC9F5-based devices. Moreover, the longer side chains in MC9F5 suppressed molecular aggregation, promoting a more favorable blend film morphology and an improved FF. As a result, the optimized MC9F5-based OSC achieved an excellent PCE of 18.02%, along with a high FF of 80.14% and a *V*_oc_ of 0.878 V. In comparison, the MC7F5-based OSC showed a lower PCE of 17.20%, with an FF of 78.85% and a *V*_oc_ of 0.832 V. Notably, both the 18.02% PCE and the 80.14% FF are among the highest values reported for OSCs that utilize ADA-type NFAs. Furthermore, MC9F5 exhibited excellent film-forming properties, making it highly suitable for large-area OSC fabrication. Encouragingly, the MC9F5-based minimodule device with an effective area of 11.09 cm^2^ showed an outstanding PCE of 15.66%, which is comparable to those of the modules that utilize Y-series acceptors (Table S1). To the best of our knowledge, MC9F5 is the first non-Y6 acceptor to achieve a PCE of >15% in a module device, demonstrating its significant potential for practical applications.

## RESULTS AND DISCUSSION

The two acceptors, MC7F5 and MC9F5, designed in this study (Fig. 1a), were synthesized by following our previously reported method [40,41], as illustrated in Schemes S1 and S2. Their molecular structures were characterized via ^1^H nuclear magnetic resonance (NMR), ^19^F NMR and high-resolution mass spectroscopy, with further details provided in the Supplementary Data. Notably, the presence of neighboring side chains in both acceptors imparts exceptional solubility in halogenated and aromatic solvents, such as dichloromethane, chloroform, toluene and chlorobenzene. The result demonstrates that a low F/H atomic ratio on the alkyl chain does not compromise the solubility of the acceptors in regular solvents, ensuring the successful material purification and device fabrication. To elucidate how the position of the –C_2_F_5_ groups on the side chain affects the molecular geometry and electronic properties, density functional theory (DFT) calculations were performed on the two NFAs at the B3LYP/6–31G** level. As illustrated in Fig. S1, both NFAs exhibit a high degree of planarity, which is conducive to achieving well-ordered intermolecular assembly and hence the intermolecular electron transport. Except for the carbonyl, cyano and halogen groups, MC7F5 and MC9F5 exhibit similar positive electrostatic potential (ESP) distributions across their π-conjugated backbones, suggesting their typical electron-accepting characteristics (Fig. 1b). Figure 1c presents the average ESP values for each atom within the fused-ring core of the two NFAs. It can be observed that the ESP values of all atoms in the fused-ring core decrease as the –C_2_F_5_ motifs move away from the conjugated backbone. This trend reveals an inverse relationship between the distance and the impact of the highly electronegative –C_2_F_5_ group on the electron density distribution across the conjugated backbone. The change in electron density distribution would further affect the electronic energy levels of the NFAs. As shown in Fig. S2, although the spatial distributions of the frontier molecular orbitals are quite similar for the two NFAs, their HOMO and LUMO energy levels are different. The calculated LUMO and HOMO energy levels for MC7F5 are –3.57 and –5.66 eV, respectively, whereas those for MC9F5 were shifted upward to –3.54 and –5.62 eV, respectively. This shift is attributed to the highly electronegative fluorine atoms that lower the charge density on the conjugated backbone through electrostatic interactions. These findings indicate that the ESP distribution and frontier orbital energy levels of NFAs can be finely tuned by varying the distance between the electronegative –C_2_F_5_ group and the π-conjugated backbone.

**Figure 2.**
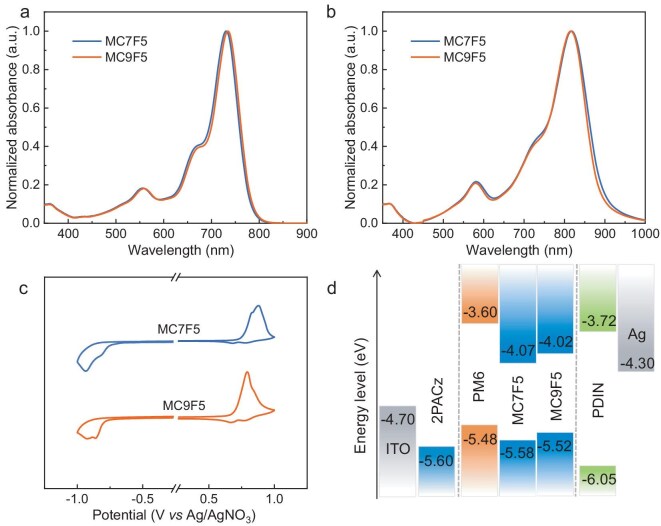
Normalized linear absorption spectra of MC7F5 and MC9F5 (a) in solutions and (b) in solid films. (c) Cyclic voltammograms of MC7F5 and MC9F5 in solid films. (d) Energy levels of MC7F5 and MC9F5 as well as other materials used for OSCs in this work.

The absorption spectra of the two acceptors were measured and compared in both diluted solutions and thin films, and the detailed photophysical data are summarized in Table S2. Due to the same molecular backbone, MC7F5 and MC9F5 exhibit similar absorption profiles in dilute chloroform solution (Fig. 2a). The absorption peak of MC7F5 is observed at 731 nm, demonstrating a blue shift of ∼4 nm in comparison with MC9F5. Such a shift is attributed to the shorter distance between the –C_2_F_5_ moieties and the molecular backbone in MC7F5 compared with that in MC9F5, which reduces the electron-donating character of the fused-ring unit in MC7F5, thereby weakening the intramolecular CT effect, as evidenced by the theoretical calculation results. The maximum extinction coefficients (*ε*_max_) for MC7F5 and MC9F5 in solution were determined to be 1.98 × 10^5^ and 2.13 × 10^5^ M^–1^ cm^–1^, respectively. The transition from solution to solid state results in bathochromic shifts in the absorption spectra of both NFAs—a phenomenon that suggests the formation of stronger intermolecular π–π interactions in the solid films. Interestingly, MC7F5 is blue-shifted in solution but red-shifted by 2 nm in solid film compared with MC9F5, which indicates stronger molecular stacking of MC7F5 than MC9F5 in the solid state. The MC9F5 film exhibits a stronger absorption band within the range of 700–900 nm, with an absorption coefficient of 2.82 × 10^5^ cm^–1^, surpassing that of the MC7F5 film, which has an absorption coefficient of 2.42 × 10^5^ cm^–1^. The optical band gaps (*E*_g_) of MC7F5 and MC9F5, determined from thin-film absorption edges, were measured at 1.36 and 1.38 eV, respectively, with both values closely approaching the ideal bandgap (1.34 eV) as defined by the Shockley–Queisser limit [42].

Cyclic voltammetry (CV) measurements were performed to investigate the electrochemical properties of MC7F5 and MC9F5, and the relevant data are summarized in Table S2. Based on the CV curves that are presented in Fig. 2c and Fig. S3, the HOMO and LUMO energy levels of MC7F5 were determined to be –5.58 and –4.07 eV, respectively. In comparison, MC9F5 exhibited slightly higher energy levels at –5.52 eV (HOMO) and –4.02 eV (LUMO), as illustrated in Fig. 2d. The variation in energy levels agrees well with the DFT calculations. Both the HOMO and LUMO energy levels of MC7F5 are downshifted compared with those of MC9F5. This phenomenon can be primarily attributed to the shorter regular alkyl chain distance between the –C_2_F_5_ moieties and the molecular backbone, which enhances electrostatic interactions and consequently reduces the electron-richness of the fused-ring core in MC7F5. Notably, reduced energy levels in the acceptor molecule would lead to increased energy offsets relative to the donor material, thereby promoting exciton dissociation and potentially enhancing *J*_sc_. However, this also leads to in an increased *E*_loss_ in the resulting OSCs.

The crystalline properties of the neat NFA films were then investigated by using grazing-incidence wide-angle X-ray scattering (GIWAXS). The 2D GIWAXS patterns, along with the corresponding line cuts, are presented in Fig. S4 and the results of the quantitative analysis are summarized in Table S3. As illustrated in the figure, despite the minimal difference of only two carbon atoms in their alkyl chain lengths, the two NFAs exhibit remarkably distinct molecular packing arrangements in the solid state. The 2D pattern of MC7F5 exhibits well-defined diffraction spots with high intensity, suggesting pronounced molecular aggregation characteristics. These structural features align well with the optical properties observed in the thin-film absorption measurements. Notably, we observed that the diffraction peaks located in the out-of-plane direction are split, which might be due to the polycrystalline texture of MC7F5. For the neat MC7F5 film, the (010) π–π diffraction peak is primarily located along the *q*_z_-axis, whereas the (100) lamellar peaks appear along the *q*_xy_-axis, indicating a preferred face-on orientation relative to the substrate. In contrast, the MC9F5 crystallites exhibit edge-on-dominated orientation, as indicated by the π–π diffraction peak along the *q*_xy_-axis and the strong (100) lamellar diffraction peak present along both the *q*_xy_- and *q*_z_-axes. Furthermore, unlike the MC7F5 film, which shows multiple narrow diffraction arcs in the out-of-plane direction beyond *q*_z_ = 0.7 Å^–1^, the MC9F5 film reveals some diffraction points at azimuthal angles between 30^o^ and 70^o^. These diffraction points are distributed similarly to the ‘satellite points’ that surround the main (100) peak, suggesting the formation of a distinct mixed polycrystalline texture with small crystalline domains [43]. The results show that, in addition to tuning the energy levels, extension of the partially fluorinated side chains can effectively restrain the excessive molecular aggregation while ensuring ordered molecular stacking, promoting the formation of a favorable nanoscale blend film morphology.

**Figure 3.**
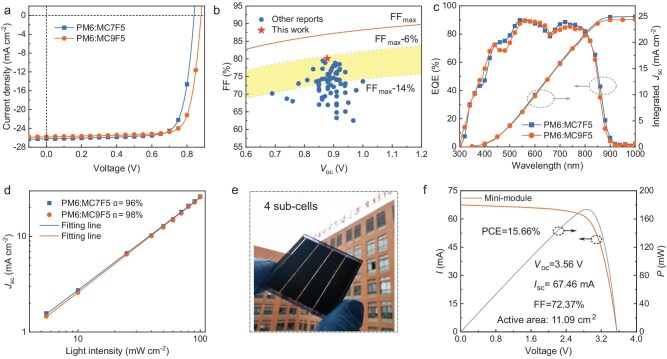
(a) *J–V* curves. (b) Plots of the *V*_oc_ versus FF for the champion OSCs based on linear ADA-type NFAs reported in this study and in recent years. The theoretically predicted lower and upper contours of FF based on empirical relationships are also shown. (c) EQE spectra and the integrated current densities from the EQE spectra of the optimal device. (d) *J*_sc_ dependence on light intensity of the optimized devices. (e) Picture of the fabricated minimodule. (f) Current–voltage (*I–V*) and power–voltage (*P–V*) characteristics of the MC9F5-based minimodule device.

To assess the photovoltaic performance of MC7F5 and MC9F5, OSCs were fabricated by using a conventional device architecture of indium tin oxide (ITO)/[2-(9H-carbazol-9-yl)ethyl] phosphonic acid (2PACz)/active layer/2,9-bis(3-(dimethylamino)propyl)anthra[2,1,9-def:6,5,10-d′e′f′]diisoquinoline-1,3,8,10(2H,9H)-tetraone (PDIN)/Ag. In this work, 2PACz was selected as the hole transport layer to minimize absorption losses and reduce contact resistance [44]. The wide-bandgap polymer PM6 (Fig. S5), which has a low-lying HOMO energy level of –5.48 eV and an absorption range from 300 to 680 nm, was chosen as the electron donor material due to its complementary absorption profile and well-matched energy levels with the two NFAs (Fig. 2d) [45]. The active layer was spin-coated from a chlorobenzene solution with chloronaphthalene (CN) as an additive. The device parameters under varying fabrication conditions are summarized in Supplementary Tables S4 and S5. The *J–V* curves and the relevant photovoltaic parameters for the optimized OSCs are provided in Fig. 3a and Table 1, respectively. The optimized device based on PM6:MC7F5 delivered a PCE of 17.20% with a *V*_oc_ of 0.832 V, a high *J*_sc_ of 26.22 mA cm^–2^ and an FF of 78.85%. To our delight, a higher PCE of 18.02% was obtained from the optimized PM6:MC9F5 binary device with a higher *V*_oc_ of 0.878 V, a *J*_sc_ of 25.62 mA cm^–2^ and an FF of 80.14%. Notably, the PCE of 18.02% for the MC9F5-based device is one of the highest values for ADA-type NFAs reported so far (Fig. S6 and Table S6). To further assess the compatibility of MC9F5 with other donor materials, we selected two commonly used polymer donors, PM1 and D18 [28], for a comprehensive investigation (Table S7). The PCEs of the resulting OSCs based on PM1:MC9F5 and D18: MC9F5 blends were 15.10% and 16.01%, respectively, both of which are lower than that of the PM6:MC9F5-based OSC. The improved photovoltaic performance of the PM6:MC9F5-based OSC in comparison with that based on PM6:MC7F5 is primarily due to its higher *V*_oc_ and FF values. This enhancement stems from the elevated CT state that reduces the *E*_loss_ within the PM6:MC9F5 device and a more balanced charge transport, as detailed in the following discussion. For comparison, we also used MC7F0 (Fig. S7), which has the regular non-fluorinated side chains, as the acceptor material to fabricate the OSCs [28]. The MC7F0-based device exhibited a *V*_oc_ of 0.88 V, which is higher than that of the MC7F5-based device but very close to that of the MC9F5-based device. This suggests that the higher *E*_loss_ caused by incorporating fluorinated side chains can be effectively mitigated by the strategy of moving the fluorinated groups on the side chains away from the conjugated backbone.

**Table 1.** Photovoltaic parameters for the PM6:MC7F5- and PM6:MC9F5-based OSCs.

Active layer	*V*_oc_ (V)	*J*_sc_ (mA cm^–^^2^)	FF (%)	PCE (%)^a^	*E*_loss_ (eV)
PM6:MC7F5	0.832	26.22	78.85	17.20 (17.14 ± 0.12)	0.639
PM6:MC9F5	0.878	25.62	80.14	18.02 (17.91 ± 0.08)	0.576

a In parentheses are average values obtained from eight devices.

The MC9F5-based devices showed remarkable FF values that are on a par with those of state-of-the-art OSCs, reflecting optimized charge transport and suppressed recombination. The maximum FF (FF_max_) without charge-transport loss can be calculated empirically by using the following equation: $F{F_{max}} = \frac{{{\gamma _{oc}} - {\mathrm{l}}n( {{\gamma _{oc}} + 0.72} )}}{{{\gamma _{oc}} + 1}}$, where *γ*_oc_ = *qV*_oc_/*nkT, q* is the elementary charge, *n* is the diode ideality factor, *k* is the Boltzmann constant and *T* is the temperature [46]. As shown in Fig. 3b, the champion FF of the optimized device reaches 80.14%, which corresponds to 92% of the FF_max_ of OSCs with a *V*_oc_ of 0.878 V. Figure 3b and Table S6 display the dependence of the FF_max_ on *V*_oc_, along with the FF values of the optimal devices from this study and previously reported OSCs based on ADA-type NFAs. Notably, the PM6:MC9F5 device achieved a higher FF value compared with other reported OSCs that utilize ADA-type NFAs. The long-term storage stability of the unencapsulated PM6:MC7F5- and PM6:MC9F5-based devices was assessed in a glove box at room temperature. As shown in Fig. S8, after ∼600 h of storage, both devices could maintain >98% of their initial PCEs, indicating a good storage stability.

Figure 3c illustrates the EQE response spectra for the two best-performing OSCs. Compared with the PM6:MC9F5-based device, the PM6:MC7F5-based device exhibited a broader photoresponse range and slightly higher EQE values, which contribute to its increased *J*_sc_ value. The integrated current densities that were derived from the EQE curves for the PM6:MC7F5 and PM6:MC9F5 devices are 24.96 and 24.38 mA cm^–2^, respectively, aligning closely with the *J*_sc_ values that were obtained from the *J–V* measurements with deviations within 5.0%. In bulk-heterojunction (BHJ) OSCs, the photon-to-electron conversion efficiency relies heavily on exciton dissociation. Accordingly, we analysed the charge dissociation probability (*P*_diss_) for the two best-performing OSCs by examining the relationship between the photocurrent density (*J*_ph_) and the effective voltage (*V*_eff_) (Fig. S9). The PM6:MC7F5-based device exhibits a higher *P*_diss_ value of 98.90% compared with 97.17% for the PM6:MC9F5-based counterpart, demonstrating enhanced exciton dissociation efficiency. This increased charge dissociation probability directly correlates with enhanced current generation in the PM6:MC7F5-based device.

The recombination kinetics in OSCs were quantitatively analysed by evaluating the dependence of *J*_sc_ on the illumination intensity (*P*_light_). The relationship between *J*_sc_ and *P*_light_ can be given by using the following equation: *J*_sc_ ∝ *P*_light_^α^, where α is the exponential factor. The α values for the PM6:MC7F5- and PM6:MC9F5-based devices, determined through linear fitting, were found to be 0.96 and 0.98, respectively (Fig. 3d). The α value closer to 1 for the PM6:MC9F5-device suggests a lower degree of charge recombination within this device. Space-charge-limited current measurements were then conducted to evaluate the charge-transport properties of the PM6:MC7F5 and PM6:MC9F5 blends. The *J–V* curves of the hole-only and electron-only devices under dark conditions are depicted in Fig. S10 and the calculated hole mobility (*μ*_h_) and electron mobility (*μ*_e_) are listed in Table S8. The *μ*_h_ and *μ*_e_ of the PM6:MC7F5 blend film were determined to be 1.09 × 10^–3^ and 5.11 × 10^–4^ cm^2^ V^–1^ s^–1^, respectively, whereas those of the PM6:MC9F5 blend film were 7.98 × 10^–4^ and 4.33 × 10^–4^ cm^2^ V^–1^ s^–1^, respectively. Consequently, the PM6:MC9F5 blend film achieved a more balanced *μ*_h_/*μ*_e_ ratio of 1.84, compared with the ratio of 2.13 for the PM6:MC7F5 blend film. More balanced charge transport and suppressed charge recombination in the PM6:MC9F5-based devices contribute to their higher FFs.

Given that the bulky partially fluorinated side chains can effectively inhibit severe molecular aggregation, we chose to use MC9F5 as the acceptor material for the fabrication of large-area devices. A photograph of the minimodule device, which consists of four subcells that are connected in series, is shown in Fig. 3e. The corresponding schematic diagram of the device structure is illustrated in Fig. S11. The measured *I*–*V* curve of the minimodule and the corresponding photovoltaic parameters are provided in Fig. 3f. This 11.09-cm^2^ module yielded a *V*_oc_ of 3.56 V, an *I*_sc_ of 67.46 mA, an FF of 72.37% and a PCE of 15.66% with respect to the active area. To the best of our knowledge, this is the first report of a module device based on a non-Y-series acceptor with an efficiency of >15% (Table S1), demonstrating the great potential of MC9F5 for fabricating large-area OSCs. Furthermore, the MC9F5-based minimodule device also exhibits good shelf stability with a low PCE loss of ∼6% after >1100 h of storage in an N_2_-filled glovebox (Fig. S12).

**Figure 4.**
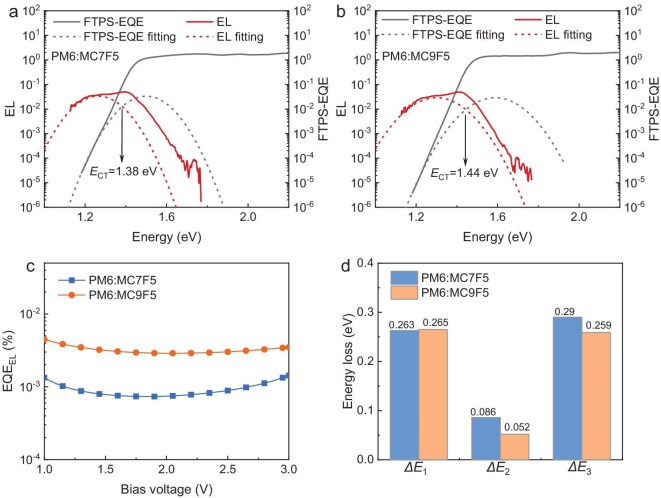
FTPS-EQE and EL spectra as well as corresponding Gaussian-fit curves via Marcus equation of the (a) PM6:MC7F5- and (b) PM6:MC9F5-based devices. (c) EQE_EL_ curves of the optimal OSCs at various bias voltages. (d) Detailed energy-loss values for the two optimal OSCs.

To investigate the difference in *V*_oc_ values between the two OSCs, we conducted a detailed analysis of the *E*_loss_ of the devices by measuring their Fourier-transform photocurrent spectroscopy external quantum efficiency (FTPS-EQE) and electroluminescence (EL) spectra. Generally, the *E*_loss_ in OSCs can be divided into three parts, as represented by the equation: *E*_loss_ = Δ*E*_1_ + Δ*E*_2_ + Δ*E*_3_ = (*E*_g_ – $qV_{{\mathrm{oc}}}^{{\mathrm{SQ}}}$) + ($qV_{{\mathrm{oc}}}^{{\mathrm{SQ}}} - qV_{{\mathrm{oc}}}^{{\mathrm{rad}}}$) + ($qV_{{\mathrm{oc}}}^{{\mathrm{rad}}} - q{V_{{\mathrm{oc}}}}$), where $qV_{{\mathrm{oc}}}^{{\mathrm{SQ}}}$ is the possible *V*_oc_ under the Shockley–Queisser limit and $qV_{{\mathrm{oc}}}^{{\mathrm{rad}}}$ represents the *V*_oc_ when considering only the radiative recombination [47]. Δ*E*_1_ is attributed to the radiative energy loss above the band gap, which is unavoidable for any solar cells. Here, the Δ*E*_1_ values for both the PM6:MC7F5- and the PM6:MC9F5-based devices are ∼0.263 eV (Table S9). The second component Δ*E*_2_ is the radiative energy loss below the band gap, which is correlated with the energy difference (Δ*E*_CT_) between the bandgap (*E*_g_^PV^) and the charge-transfer state (*E*_CT_) of the OSC devices. The *E*_g_^PV^ values were 1.45 and 1.47 eV for the MC7F5- and MC9F5-based devices, respectively, as determined by using the cross point of the normalized emission and the absorption of the neat acceptor films (Fig. S13). The *E*_CT_ values of the MC7F5- and MC9F5-based devices can be obtained by fitting FTPS-EQE and EL spectra. As shown in Fig. 4a and b, the *E*_CT_ of the MC7F5-based OSC is determined to be 1.38 eV, whereas that of the MC9F5-based OSC is 1.44 eV. Correspondingly, the Δ*E*_CT_ value decreases from 0.07 to 0.03 eV, which is likely to be the primary factor that leads to the relatively lower *P*_diss_ and reduced *J*_sc_ observed in the MC9F5-based device. Moreover, changes in the Δ*E*_CT_ values also lead to variations in Δ*E*_2_, as presented in Fig. 4d, in which the Δ*E*_2_ values for the MC7F5- and MC9F5-based devices are 0.086 and 0.052 eV, respectively. Δ*E*_3_ is the non-radiative recombination loss and can be quantified as (–*k*T/*q*)ln(EQE_EL_), where *k* is the Boltzmann constant, *T* is the Kelvin temperature and EQE_EL_ is the electroluminescence quantum efficiency of the device. It is generally assumed that lowering the Δ*E*_CT_ of the OSC facilitates hybridization between the LE and CT states, which can enhance the luminescence of the CT state by borrowing the highly luminescent LE state [36]. Also, a smaller Δ*E*_CT_ facilitates the transition from the CT state back to the LE state, thus allowing the CT state to undergo radiative recombination through the emissive LE state. In any case, enhancing the luminescence of the CT state can effectively reduce the non-radiative recombination loss (i.e. Δ*E*_3_) of the OSC. To confirm this mechanism and quantitatively calculate the Δ*E*_3_ value, the EQE_EL_ spectra of the two OSCs were measured. As shown in Fig. 4c, the MC7F5-based device exhibits an EQE_EL_ of 9.19 × 10^–6^, corresponding to a Δ*E*_3_ of 0.290 eV whereas the MC9F5-based device shows a higher EQE_EL_ of 3.17 × 10^–5^, leading to a lower Δ*E*_3_ of 0.259 eV. Based on the above data, the *E*_loss_ of the MC9F5-based device is calculated to be 0.576 eV, which is considerably lower than the *E*_loss_ of 0.639 eV observed for the MC7F5-based device. The results indicate that the significant improvement in *V*_oc_ of the MC9F5 device can be attributed to the elevated *E*_CT_ induced by the increased LUMO energy level, which leads to the simultaneous reduction of radiative and non-radiative recombination losses.

**Figure 5.**
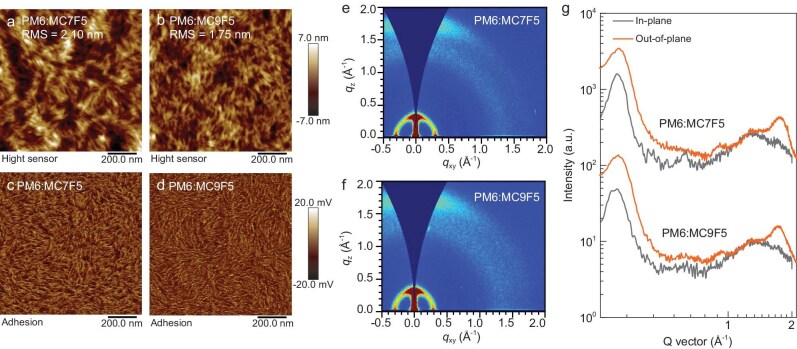
(a, b) AFM height and (c, d) phase images of the optimized PM6:MC7F5 and PM6:MC9F5 blend films. (e, f) Two-dimensional GIWAXS patterns and (g) in-plane and out-of-plane line cuts of the PM6:MC7F5 and PM6:MC9F5 blend films.

The top-surface morphology of the MC7F5- and MC9F5-based BHJ blend films was examined by using atomic force microscopy (AFM) in a peak force quantitative nanomechanical mapping mode. As shown in Fig. 5a and b, both blend films display a fibrillar network morphology, which is beneficial for efficient charge transport. The root-mean-square roughness values under optimal conditions are 2.10 nm for the MC7F5-based film and 1.75 nm for the MC9F5-based film. The higher roughness value in the MC7F5-based film is attributed to its stronger molecular aggregation. AFM phase images (Fig. 5c and d) reveal phase separation in both blends; however, the PM6:MC7F5 blend film shows more pronounced phase separation and relatively larger phase domains compared with the PM6:MC9F5 blend film. The larger phase domains in the PM6:MC7F5 blend film may promote charge recombination, thereby adversely affecting the FF of the OSC devices. GIWAXS was subsequently used to analyse the microstructures and molecular packing behaviors of the blend films. As shown in Fig. 5e and f, both blend films exhibit distinct π–π stacking diffraction peaks along the *q*_z_-axis and lamellar stacking diffraction peaks along the *q*_xy_-axis, indicating the formation of a preferential face-on orientation. For the π–π stacking diffraction in the *q*_z_-axis, the PM6:MC9F5 blend film exhibits a strong diffraction peak at 1.73 Å^–1^, corresponding to a π–π stacking distance (*d*_π__–__π_) of 3.63 Å, with a coherent length (CL) of 16.44 Å (Table S3). In comparison, the (010) diffraction peak of the PM6:MC7F5 blend film is slightly shifted to 1.75 Å^–1^, giving a slightly shorter *d*_π__–__π_ of 3.59 Å with an enhanced CL of 18.85 Å. The enhanced crystallinity of the PM6:MC7F5 blend film is favorable for achieving a higher *J*_sc_ value in the resulting devices.

## CONCLUSION

In conclusion, we have successfully designed, synthesized and characterized two M-series NFAs, namely MC7F5 with 8,8,9,9,9-pentafluoro-2-(6,6,7,7,7-pentafluoroheptyl)nonyl side chains and MC9F5 with 10,10,11,11,11-pentafluoro-2-(8,8,9,9,9-pentafluorononyl)undecyl side chains, for applications in OSCs. Extending the length of the partially fluorinated and branched side chains in MC9F5 effectively suppresses molecular aggregation, resulting in blend films with an optimal nanoscale phase separation morphology that is characterized by small domain sizes and good crystallinity. This favorable morphology facilitates balanced charge transport and reduces bimolecular recombination, yielding an impressive FF of 80.14% in the MC9F5-based devices, surpassing that of the MC7F5-based devices. Furthermore, positioning the fluorinated –C_2_F_5_ groups farther from the conjugated backbone diminishes the impact of the strong electronegativity of the fluorine atoms on the electronic properties of the resulting MC9F5, raising its LUMO energy level as well as the *E*_CT_ of the resulting blend film. The elevated *E*_CT_ reduces both radiative and non-radiative recombination losses, thereby improving the *V*_oc_ of MC9F5-based devices. Consequently, the optimized OSC based on PM6:MC9F5 achieved a remarkable PCE of 18.02%, which is one of the highest reported values for ADA-type fused-ring NFAs. In contrast, the PM6:MC7F5-based device delivered a moderate PCE of 17.20%, limited by the lower *V*_oc_ and FF values. Notably, MC9F5, with its favorable aggregation property, is also suitable for large-area device fabrication, achieving an excellent PCE of 15.66% in a minimodule device with an active area of 11.09 cm². Our findings demonstrate that fine-tuning the spatial separation between the fluorinated moieties of the side chain and the conjugated backbone allows precise modulation of both the LUMO energy levels and the *E*_CT_ characteristics, while maintaining optimal blend film morphology. This molecular engineering approach offers a promising design strategy for developing high-performance ADA-type acceptor materials.

## Supplementary Material

nwaf089_Supplemental_File
