# Supplementary material for: Photovoltage enhancement of M-series acceptor-based polymer solar cells and minimodules through the modulation of charge-transfer states
Source: Natl Sci Rev. 2025 Mar 7;12(5):nwaf089. doi: 10.1093/nsr/nwaf089 (PMC11970251; doi:10.1093/nsr/nwaf089)
Supplement: nwaf089_Supplemental_File [file nwaf089_supplemental_file.pdf]

Supplementary data

Photovoltage Enhancement of M-Series Acceptor-Based Polymer Solar Cells and Minimodules through the Modulation of Charge Transfer States

Kaichen Xing[1,3,4], Dongdong Cai[1], Di Wang[1], Jin-Yun Wang[1], Changquan Tang[1,4], Yunlong Ma[1,4,*] and Qingdong Zheng[2,*]

[1]State Key Laboratory of Structure Chemistry, Fujian Institute of Research on the Structure of Matter, Chinese Academy of Sciences, Fuzhou 350002, China.

[2]State Key Laboratory of Coordination Chemistry, College of Engineering and Applied Sciences, Nanjing University, Nanjing 210023, China.

[3]College of Chemistry, Fuzhou University, Fuzhou 350116, China

[4]Fujian College, University of Chinese Academy of Sciences, Fuzhou 350002, China

[*]Corresponding author.

E-mail: mayunlong@fjirsm.ac.cn; zhengqd@nju.edu.cn

1. Materials and instruments

PM6 and 2-(5,6-difluoro-3-oxo-2,3-dihydro-1H-inden-1-ylidene)malononitrile (IC2F) were purchased from Solarmer Materials Inc. and Suna Tech Inc., respectively. Other solvents and reagents were purchased from Aldrich Inc., Adamas-beta Ltd., Suna Tech Inc. and Energy Chemical, and used directly without further purification unless otherwise specified. ^{1}H NMR and ^{19}F NMR spectra were recorded on a Bruker AVANCE-400 spectrometer using tetramethylsilane (TMS) as the internal standard. High-resolution mass spectroscopy measurements were recorded by using a UHR TOF LC/MS Mass Spectrometer. Absorption spectra were obtained from a Lambda 365 UV-vis spectrophotometer. Surface morphology of the blend film was tested using atomic force microscopy (AFM) in a Peak Force Quantitative Nanomechanical Mapping mode. Cyclic voltammetry (CV) measurement was performed on a CHI 604E electrochemical workstation with a three-electrode cell in a nitrogen-bubbled 0.1 M tetrabutylammonium hexafluorophosphate (Bu_4NPF_6) solution in acetonitrile at a scan rate of 100 mV s^{-1} at room temperature. Platinum wire, Ag/$AgNO_3$ (0.1 M $AgNO_3$ in acetonitrile), and platinum plate were used as the counter electrode, reference electrode, and working electrode, respectively. The Ag/$AgNO_3$ reference electrode was calibrated using a ferrocene/ferrocenium redox couple as an external standard, whose oxidation potential is set at -4.82 eV with respect to zero vacuum level. The acceptor films were coated on the Pt plate electrode by dipping the electrode into corresponding solutions and then drying. The HOMO and LUMO energy levels of the materials were calculated according to the equations of $E_{\mathrm{HOMO}} = -(\varphi_{\mathrm{ox}} + 4.82)$ (eV) and $E_{\mathrm{LUMO}} = -(\varphi_{\mathrm{red}} + 4.82)$ (eV), respectively, where φ_{ox} and φ_{red} are the onset oxidation and reduction potentials.

2. **Synthesis and characterization**

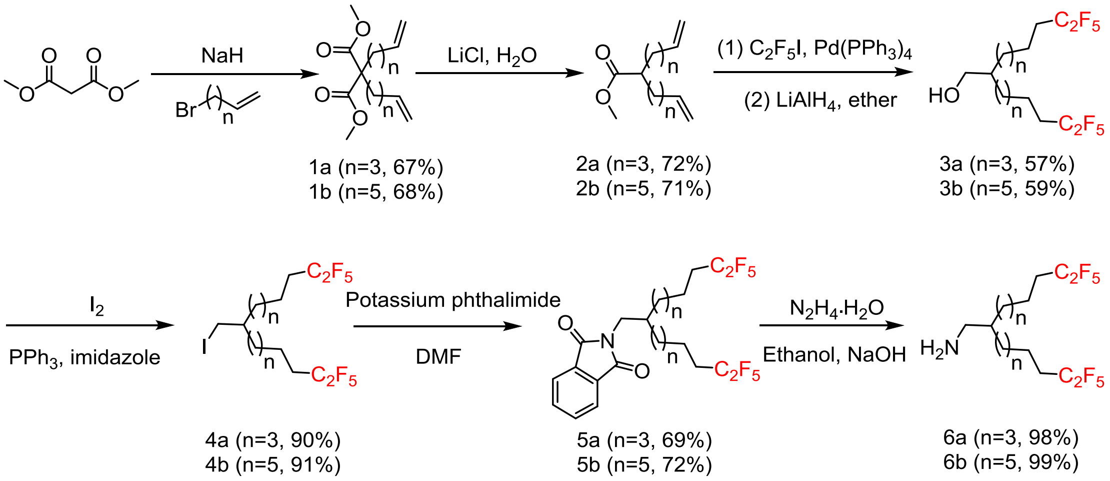

Scheme S1. The synthetic route of partially fluorinated side-chains 6a and 6b.

Synthesis of Compound 1a: To a two necked flask, NaH (60 wt%) 16.70 g was added to a solution of dimethyl malonate (18.38 g, 139 mmol) in DMF at 0 ℃ under nitrogen atmosphere. After 1 h, 5-bromo-1-pentene (49.78 g, 334 mmol) was added dropwise. The reaction mixture was stirred overnight. After the reaction, the mixture was poured into water and then extracted with petroleum ether. The combined organic layer was washed with water and dried over anhydrous $MgSO_4$. The solvent was removed under reduced pressure and the crude product was purified by silica gel column using petroleum ether/dichloromethane (2:1 by volume) as the eluent, yielding compound 1 as a colorless oil (26.5 g, 67%). ^{1}H NMR (400 MHz, $CDCl_3$, δ): 5.84-5.66 (m, 2H), 5.05-4.86 (m, 4H), 3.70 (s, 6H), 2.09-1.99 (m, 4H), 1.94-1.82 (m, 4H), 1.30-1.18 (m, 4H).

Synthesis of Compound 1b: Compound 1b was synthesized according to the same procedure as that for 1a. Compound 1b was isolated as a yellow oil in 68% yield. ^{1}H NMR (400 MHz, $CDCl_3$, δ): 5.88-5.68 (m, 2H), 5.07-4.82 (m, 4H), 3.70 (s, 6H), 2.09-1.96 (m, 4H), 1.92-1.79 (m, 4H), 1.44-1.23 (m, 8H), 1.19-1.06 (m, 4H).

Synthesis of Compound 2a: To a round bottom flask, a mixture of compound 1a (26.5g, 98.79 mmol), LiCl (8.38 g, 197.58 mmol) and water (1.96 g, 108.89 mmol) in DMSO (220 mL) was heated at refluxing for 6 h. After cooling to room temperature,

the mixture was poured into water and extracted with petroleum ether. The combined organic layers were washed with water and dried over anhydrous $MgSO_4$. After the evaporation of solvent, the residue was purified by column chromatography with petroleum ether/dichloromethane (2:1 by volume) as eluent to yield compound 2a as a colorless liquid (14.7 g, 72%). ^{1}H NMR (400 MHz, $CDCl_3$, δ): 5.83-5.70 (m, 2H), 5.03-4.83 (m, 4H), 3.67 (s, 3H), 2.40-2.25 (m 1H), 2.11-1.93 (m, 4H), 1.66-1.52 (m, 4H), 1.50-1.17 (m, 4H).

Synthesis of Compound 2b: Compound 2b was synthesized according to the same procedure as that for 2a. Compound 2b was isolated as a yellow oil in 71% yield. ^{1}H NMR (400 MHz, $CDCl_3$, δ): 5.85-5.69 (m, 2H), 5.05-4.87 (m, 4H), 3.67 (s, 3H), 2.39-2.26 (m 1H), 2.08-1.98 (m, 4H), 1.61-1.53 (m, 2H), 1.45-1.38 (m, 2H), 1.38-1.31 (m, 4H), 1.31-1.19 (m, 8H).

Synthesis of Compound 3a: A clean, oven-dried 300 mL screw cap Schlenk reaction tube with magnetic stir bar was charged with $Pd(PPh_3)_4$ (3.63 g, 3.14 mmol) and compound 2a (22 g, 104.6 mmol). The tube was then evacuated and back-filled with nitrogen. Under a counter flow of nitrogen, pentafluoroethyl iodide (77.17 g, 313.80 mmol) and n-hexane (150 mL) were added sequentially. The tube was tightly closed by screw cap and placed in room temperature. The reaction mixture was stirred for 48 h. The solvent was removed under reduced pressure and the crude product was purified by silica gel column using petroleum ether/dichloromethane (2:1 by volume) as the eluent. Then, the product (colorless liquid) was taken up in dry ethyl ether Et_2O (30 mL). Under nitrogen atmosphere, the mixed solution was added dropwise to a suspension of $LiAlH_4$ (9.94 g, 261.51 mmol) in dry Et_2O (70 mL) at a rate that maintain the solution reflux. After dropwise addition, the mixture was heated to reflux for additional 4 h. A 30 wt% aqueous H_2SO_4 was then added to dissolve all the precipitated solids. The aqueous layer was extracted with EtOAc. The organic layer was separated and washed with water and brine, and dried over anhydrous $MgSO_4$. After the removal of solvent, the residue was purified by column chromatography on silica gel using petroleum ether/dichloromethane (1:1) as the eluent, yielding

compound 3a as a colorless liquid (25 g, 57%). ^{1}H NMR (400 MHz, $CDCl_3$, δ): 3.54 (d, *J*=5.40 Hz, 2H), 2.10-1.89 (m, 4H), 1.68-1.52 (m, 4H),1.52-1.44 (m, 1H) ,1.44-1.19 (m, 12H). ^{19}F NMR (376 MHz, $CDCl_3$, δ): -85.48 (s), -118.29 (s).

Synthesis of Compound 3b: Compound 3b was synthesized according to the same procedure as that for compound 3a. Compound 3b was isolated as a yellow oil in 59% yield. ^{1}H NMR (400 MHz, $CDCl_3$, δ): 3.54 (d, *J*=5.40 Hz, 2H), 2.09-1.91(m, 4H), 1.64-1.52 (m, 4H),1.49-1.42 (m, 1H) ,1.43-1.19 (m, 20H). ^{19}F NMR (376 MHz, $CDCl_3$, δ): -85.47 (s), -118.30 (s).

Synthesis of Compound 4a: To a round bottom flask, iodine (9.63 g, 37.95 mmol) was added to a solution of Compound 3 (12.33 g, 29.19 mmol), triphenylphosphine (9.95 g, 37.95 mmol), and imidazole (2.58 g, 37.95 mmol) in 150 mL of dichloromethane at 0 ℃. After stirring for 15 min, the reaction mixture was allowed to warm to room temperature, and stirred overnight under dark conditions. The solvent was removed under reduced pressure and the crude product was purified by silica gel column using petroleum ether as the eluent, yielding compound 4a as a colorless liquid (14 g, 90%). ^{1}H NMR (400 MHz, $CDCl_3$, δ): 3.26 (d, *J*=4.48 Hz, 2H), 2.10-1.93 (m, 4H), 1.66-1.55 (m, 4H), 1.44-1.21 (m, 13H). ^{19}F NMR (376 MHz, $CDCl_3$, δ): -85.44 (s), -118.23 (s).

Synthesis of Compound 4b: Compound 4b was synthesized according to the same procedure as that for 4a. Compound 4b was isolated as a yellow oil in 91% yield. ^{1}H NMR (400 MHz, $CDCl_3$, δ): 3.27 (d, *J*=4.48 Hz, 2H), 2.08-1.93 (m, 4H), 1.65-1.50 (m, 4H) ,1.43-1.19 (m, 21H). ^{19}F NMR (376 MHz, $CDCl_3$, δ): -85.44 (s), -118.27 (s).

Synthesis of Compound 5a: In a dry two-neck round-bottomed flask, compound 4a (14 g, 26.30 mmol) was dissolved in 100 mL of DMF and followed by addition of potassium phthalimide (7.31 g, 39.46 mmol). The mixture was stirred for 72 h at 25 ℃. After the reaction, the mixture was poured into water and then extracted with petroleum ether. The combined organic layer was washed with water and brine, dried over anhydrous $MgSO_4$. After the removal of solvent, the residue was purified by

column chromatography on silica gel using petroleum ether/dichloromethane (1:1) as the eluent, yielding Compound 5a as a colorless oil (10 g, 69%). ^{1}H NMR (400 MHz, $CDCl_3$, δ): 7.85 (dd, J_1= 5.52 Hz, J_2=3.12 Hz, 2H), 7.73 (dd, J_1= 5.52 Hz, J_2=3.12 Hz, 2H), 3.57 (d, J=7.12 Hz, 2H), 2.08-1.91 (m, 4H), 1.91-1.82 (m, 1H), 1.64-1.50 (m, 4H), 1.47-1.20 (m, 12H). ^{19}F NMR (376 MHz, $CDCl_3$, δ): -85.44 (s), -118.27 (s).

Synthesis of Compound 5b: Compound 5b was synthesized according to the same procedure as that for compound 5a. Compound 5b was isolated as a yellow oil in 72% yield. ^{1}H NMR (400 MHz, $CDCl_3$, δ): 7.84 (dd, J_1= 5.44 Hz, J_2=3.08 Hz, 2H), 7.73 (dd, J_1= 5.44 Hz, J_2=3.08 Hz, 2H), 3.57 (d, J=7.16 Hz, 2H), 2.08-1.91 (m, 4H), 1.91-1.83 (m, 1H), 1.61-1.50 (m, 4H), 1.42-1.21 (m, 20H). ^{19}F NMR (376 MHz, $CDCl_3$, δ): -85.47 (s), -118.29 (s).

Synthesis of Compound 6a: Compound 5a (10 g, 17.14 mmol) and 6 mL of hydrazine hydrate (85 wt% solution in H_2O) were added to 100 mL of ethanol, and the mixture was refluxed overnight. The resulting precipitate was collected by filtration and then dissolved in 100 mL of water. The resulting solution was made alkaline by adding 6 M aqueous NaOH. After that, the mixture was extracted with petroleum ether. The combined organic layer was washed with water and brine, and dried over anhydrous $MgSO_4$. After the removal of solvent, compound 6a was obtained as a colorless liquid (7.15 g, 98%). ^{1}H NMR (400 MHz, $CDCl_3$, δ): 2.62 (d, J=3.84 Hz, 2H), 2.09-1.92 (m, 4H), 1.64-1.54 (m, 4H), 1.43-1.20 (m, 13H). ^{19}F NMR (376 MHz, $CDCl_3$, δ): -85.58 (s), -118.39 (s).

Synthesis of Compound 6b: Compound 6b was synthesized according to the same procedure as that for 6a. Compound 6b was isolated as a yellow oil in 99% yield. ^{1}H NMR (400 MHz, $CDCl_3$, δ): 2.59 (d, J=4.04 Hz, 2H), 2.07-1.90 (m, 4H), 1.63-1.50 (m, 4H), 1.42-1.06 (m, 21H). ^{19}F NMR (376 MHz, $CDCl_3$, δ): -85.39 (s), -118.22 (s).

The tetrabrominated derivative (compound 7) was synthesized according to the method previously reported by our group.[1]

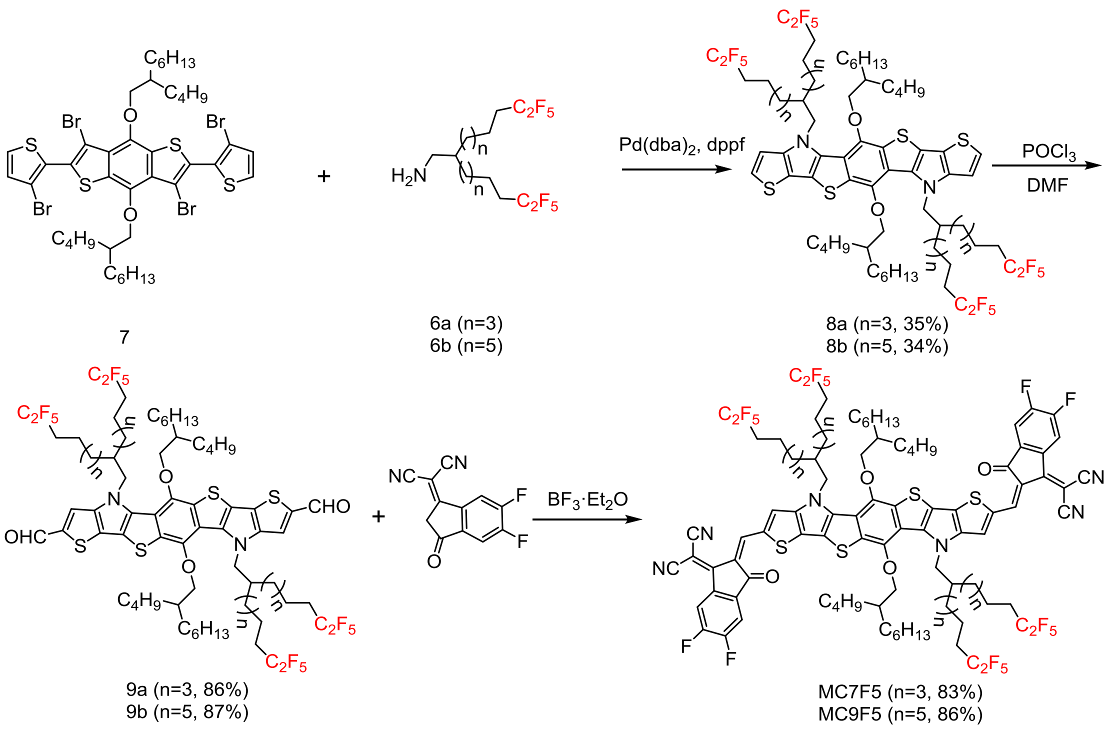

Scheme S2. The synthetic route for MC7F5 and MC9F5.

Synthesis of Compound 8a: To a suspension of sodium *tert*-butoxide (4.64 g, 2.42 mmol) in 15 mL of anhydrous toluene were added compound 7 (2.51 g, 2.50 mmol), $Pd(dba)_2$ (0.14 g, 0.24 mmol) and dppf (0.54 g, 0.97 mmol). The resulting mixture was deoxygenated with nitrogen for 30 min, and then, Compound 6a (3.06 g, 7.26 mmol) was added. The mixture was stirred at reflux for 12 h under nitrogen. After cooling to room temperature, the mixture was poured into deionized water and extracted with dichloromethane. The combined organic phase was washed with water and dried with $MgSO_4$. After evaporation of solvent, the residue was purified by column chromatography with petroleum ethers as eluent yielding compound 8a as a light-yellow oil (1.23 g, 35%). ^{1}H NMR (400 MHz, $CDCl_3$, δ): 7.23 (d, *J* = 5.24 Hz, 2H), 7.07 (d, *J*= 5.24 Hz, 2H), 4.69 (d, *J*= 7.56 Hz, 4H), 3.99 (d, *J*= 7.00 Hz, 4H) , 2.14-1.76(m, 12H), 1.71-0.81 (m, 76H); ^{19}F NMR (376 MHz, $CDCl_3$, δ): -85.47 (s) , -118.29 (s); HRMS (MALDI) m/z: calcd. for $C_{74}H_{100}F_{20}N_2O_2S_4$, 1556.6343; found, 1556.6293.

Synthesis of Compound 8b: Compound 8b was synthesized according to the same procedure as that for compound 8a. Compound 8b was isolated as a yellow oil

in 34% yield. ^{1}H NMR (400 MHz, $CDCl_3$, δ): 7.21 (d, *J*= 5.20 Hz, 2H), 7.08 (d, *J*= 5.20 Hz, 2H), 4.71 (d, *J*= 7.52 Hz, 4H), 3.99 (d, *J*= 6.92 Hz, 4H) , 2.17-1.82 (m, 12H), 1.73-0.81 (m, 92H); ^{19}F NMR (376 MHz, $CDCl_3$, δ): -85.47 (s), -118.25 (s); HRMS (MALDI) m/z: calcd. for $C_{82}H_{116}F_{20}N_2O_2S_4$, 1668.7595; found, 1668.7544.

Synthesis of Compound 9a: In a dry two neck round-bottomed flask, compound 9a (1.23 g, 0.79 mmol) was dissolved in 35 mL of 1,2-dichloroethane and placed under nitrogen atmosphere. The solution was cooled to 0 ℃ and stirred while phosphorus oxychloride (2.42 g, 15.79 mmol) and DMF (1.21 g, 16.58 mmol) were added successively. The mixture was stirred for 1 h at 0 ℃, and then stirred for 12 h at 60 ℃. After the reaction, the mixture was cooled to room temperature and poured into ice water, neutralized with Na_2CO_3, and then extracted with dichloromethane. The combined organic layer was washed with water and brine, dried over anhydrous $MgSO_4$. After the removal of solvent, the residue was purified by column chromatography on silica gel using petroleum ether/dichloromethane (1:1) as the eluent, yielding compound 9a as an orange crystalline solid (1.09 g, 86%). ^{1}H NMR (400 MHz, $CDCl_3$, δ): 9.95 (s, 2H), 7.72 (s, 2H), 4.76 (d, *J*=7.60 Hz, 4H), 4.02 (d, *J*=6.96 Hz, 4H), 2.25-1.76 (m, 12H) , 1.70-0.80 (m, 76H); ^{19}F NMR (376 MHz, $CDCl_3$, δ): -85.47 (s), -118.26 (s); HRMS (MALDI) m/z: calcd. for $C_{76}H_{100}F_{20}N_2O_4S_4$, 1612.6241; found, 1612.6200.

Synthesis of Compound 9b: Compound 9b was synthesized according to the same procedure as that for compound 9a. Compound 9b was isolated as a yellow oil in 87% yield. ^{1}H NMR (400 MHz, $CDCl_3$, δ): 9.95 (s, 2H), 7.72 (s, 2H), 4.76 (d, *J* = 7.60 Hz, 4H), 4.00 (d, *J* = 6.96 Hz, 4H), 2.25-1.82 (m, 12H), 1.72-0.80 (m, 92H); ^{19}F NMR (376 MHz, $CDCl_3$, δ): -85.47 (s), -118.26 (s); HRMS (MALDI) m/z: calcd. for $C_{84}H_{116}F_{20}N_2O_4S_4$, 1724.7493; found, 1724.7451.

Synthesis of MC7F5: Compound 9a (0.06 g, 0.037 mmol) and 2-(5,6-difluoro-3-oxo-2,3-dihydro-1H-inden-1-ylidene)malononitrile (0.02 g, 0.087 mmol) were dissolved in 35 mL of toluene, and then acetic anhydride (0.06 mL) and boron trifluoride etherate (0.05 g, 0.35 mmol) were added slowly under stirring. The

resulting mixture was stirred at room temperature for 0.5 h under N_2 atmosphere. After that, the reaction mixture was poured into methanol and the precipitate was filtered off. The crude product was then purified by silica gel column using petroleum ether/dichloromethane (1:1 by volume) as the eluent. The target acceptor (MC7F5) was obtained as a dark blue crystalline solid (0.063 g, 83%). ^{1}H NMR (400 MHz, $CDCl_3$, δ): 8.95 (s, 2H), 8.54 (dd, J_1= 10.04, J_2=6.48 Hz, 2H), 7.96 (s, 2H), 7.70 (t, J= 7.52 Hz, 2H), 4.75 (d, J= 7.64 Hz, 4H), 4.03 (d, J= 6.96 Hz, 4H), 2.24-0.80 (m, 88H); ^{19}F NMR (376 MHz, $CDCl_3$, δ): -85.48 (s), -118.23 (s); -122.96 (d, J=19.18 Hz), -124.10 (d, J= 19.18 Hz); HRMS (MALDI) m/z: calcd. for $C_{100}H_{104}F_{24}N_6O_4S_4$, 2036.6613; found, 2036.6569.

Synthesis of MC9F5: MC9F5 was synthesized according to the same procedure as that for MC7F5. A dark blue crystalline solid of MC9F5 was isolated in 86% yield. 1H NMR (400 MHz, $CDCl_3$, δ): 8.96 (s, 2H), 8.54 (dd, J_1= 9.76 Hz, J_2=6.24 Hz, 2H), 7.88 (s, 2H), 7.69 (t, J= 7.48 Hz, 2H), 4.75 (d, J= 7.64 Hz, 4H), 4.02 (d, J= 6.92 Hz, 4H), 2.25-0.76 (m, 104H); ^{19}F NMR (376 MHz, $CDCl_3$, δ): -85.49 (s), -118.28 (s); -123.07 (d, J= 19.44 Hz), -124.20 (d, J= 19.44 Hz); HRMS (MALDI) m/z: calcd. for $C_{108}H_{120}F_{24}N_6O_4S_4$, 2148.7865; found, 2148.7825.

3. OSCs fabrication and characterization

Small-area device fabrication: OSC devices were fabricated with a device structure of ITO (15 Ω)/2PACz/active layer/PDIN/Ag. ITO-coated glass substrates were cleaned by ultrasonicating sequentially in detergent, deionized water, acetone, and isopropanol for 10 min each and then dried in an oven at 80 ^{o}C for 12 h. Then, the ITO glass substrates were subjected to ultraviolet/ozone treatment at room temperature for 15 min. The self-assembled monolayer of 2PACz was prepared according to the method published by Lin *et al.*[2] 2PACz-modified substrates were then transferred into a N_2-filled glovebox for spin-coating the active layer. For the active-layer film, the PM6: NFA blend with a ratio of 1:1 was dissolved in chloroform solution (20 mg/mL) with 0.5% CN (v/v) and then spin-coated at 1500 rpm for 30 s.

All the films were thermally annealed at 80 ℃ for 5 min. Successively, PDIN methanol solution (2.0 mg/mL) was spin-coated on the active layer at 3000 rpm for 30 s to afford a buffer layer. Finally, 100 nm of Ag top electrode was deposited onto the PDIN buffer layer through shadow masks by thermal evaporation at a pressure of 1.0×10^{-4} Pa. The active area of the devices was 4.15 mm^2.

Large-area module fabrication: The fabrication conditions for the large-area OSC modules were largely identical to those used for small-area devices, except for two key differences: the device structure was modified to ITO (10 Ω)/PEDOT:PSS/active layer/ PDIN/Ag, and a mechanical scribe with a pointed cotton swab was employed to create the P2 patterns in this work.

The current density-voltage (*J*-*V*) characteristics were measured using a Keithley 2400 Source-Measure Unit. An Oriel Sol3A simulator (Newport) was used as a light source. The light intensity was calibrated to 100 mW cm^{-2} by a NREL certified silicon reference cell. EQE data were taken by using the QE/IPCE measurement kit (QE-PV-SI) from Newport.

4. Hole- and electron-only device fabrication and characterization

Hole- and electron- mobilities were measured using the space charge limited current (SCLC) method. Hole-only devices were fabricated with a architecture of ITO/PEDOT:PSS/active layer/MoO_3/Ag, while electron-only devices were constructed with a architecture of ITO/ZnO/active layer/PDIN/Al. The active layers were prepared using the same method as that used for the best-performance OSC fabrication. Device areas were fixed at 4.15 mm^2. The current density (J) was measured by a Keithley 2400 source measurement unit. The SCLC hole/electron mobilities were calculated according to the following equation:

$$J = \frac{9\varepsilon_0\varepsilon_r\mu V^2}{8L^3} \qquad (1)$$

Where J is the current density (A m^{-2}), ε_0 is the free-space permittivity (8.85×10^{-12} F m^{-1}), ε_r is the relative dielectric constant of the active layer material usually 2-4 for organic semiconductors, herein we used a relative dielectric constant of 3, μ is

the mobility of hole or electron, V is the voltage drop across the SCLC device ($V = V_{app}-V_{bi}$, where V_{app} is the applied voltage to the device and V_{bi} is the built-in voltage due to the difference in the work function of two electrodes, in the hole- and electron-only devices, the V_{bi} values are 0.5 and 0.7 V, respectively), and L is the thickness of the active layer. The film thickness was determined by a Bruker Dektak XT surface profilometer. The hole- or electron-mobilities were calculated from the slopes of the $J^{1/2}$-V curves.

5. GIWAXS characterization

All samples for GIWAXS measurements were prepared on the PEDOT: PSS-coated Si substrates using the same method as that used for the best-performance OSCs fabrication. The 2D GIWAXS patterns were acquired using an XEUSS SAXS/WAXS system at the Fujian Science & Technology Innovation Laboratory for Optoelectronic Information of China. The wavelength of the X-ray beam is 1.54 Å, and the incident angle was set as 0.2°. Scattered X-rays were detected by using a Dectris Pilatus 300 K photon counting detector.

6. Highly sensitive EQE and EQE_{EL} measurements

Highly sensitive EQE (s-EQE) was measured using an integrated system (PECT-600, Enlitech). External quantum efficiency of electroluminescence (EQE_{EL}) and electroluminescence (EL) spectra were collected by applying external voltage (1-3 V) through the devices (ELCT-3010, Enlitech). All devices for EQE_{EL} measurements were prepared according to the optimal device fabrication conditions.

7. Calculation of the energy loss

Devices for EQE_{EL} and EL spectral measurements were prepared based on optimized conditions. The detailed calculation of energy loss is as follows:

$$V_{oc}^{SQ} = \frac{kT}{q}\ln\left(\frac{J_{SC}}{J_0^{SQ}}+1\right) = \frac{kT}{q}\ln\left(\frac{q\cdot\int_0^{\infty} EQE_{PV}(E)\cdot\Phi_{AM1.5}(E)dE}{q\cdot\int_{E_{gap}}^{\infty}\Phi_{BB}(E)dE}+1\right) \quad (2)$$

$$V_{oc}^{rad} = \frac{kT}{q}\ln\left(\frac{J_{SC}}{J_0^{rad}}+1\right) = \frac{kT}{q}\ln\left(\frac{q\cdot\int_0^{\infty} EQE_{PV}(E)\cdot\Phi_{AM1.5}(E)dE}{q\cdot\int_0^{\infty} EQE_{PV}(E)\cdot\Phi_{BB}(E)dE}+1\right) \quad (3)$$

$$\Phi_{BB}(E) = \frac{2\pi}{h^3c^2}E^2\exp(-\frac{E}{KT}) \quad (4)$$

$$\Delta E_1 = E_g\text{-}V_{oc}^{SQ} \quad (5)$$

$$\Delta E_2 = V_{oc}^{SQ} - V_{oc}^{rad} \quad (6)$$

$$\Delta E_3 = \text{-}\frac{kT}{q}\ln EQE_{EL} \quad (7)$$

Where q is the elementary charge; V_{oc}^{SQ} is the maximum voltage in the Shockley-Queisser (SQ) limit model, and V_{oc}^{rad} is the V_{oc} with only radiative recombination in the device. The integral boundaries a and b are selected where P(a) = P(b) = 0.5max[P(E_g)]. The selection of integral boundaries serves to exclude the influence of noisy data and negative value of P(E_g), and is not physically motivated. While the factor 0.5 in the choice of a and b is fairly arbitrary, slightly different choices would not strongly affect the result except for very noisy data.[3]

8. Additional figures and tables

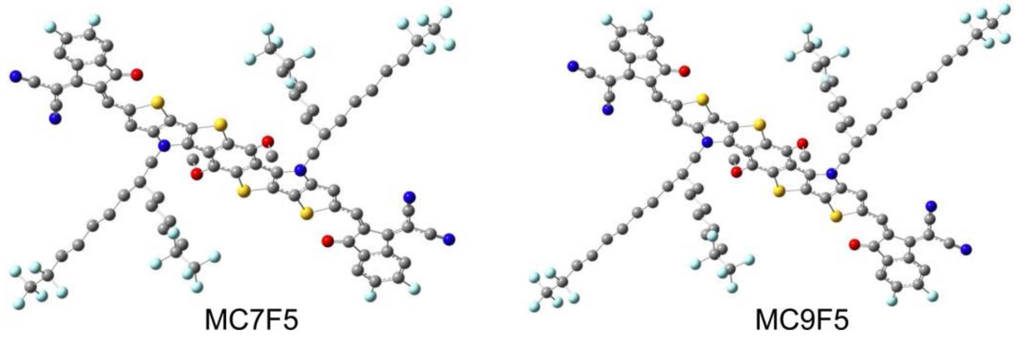

Figure S1. Optimal geometries of MC7F5 and MC9F5 from side view.

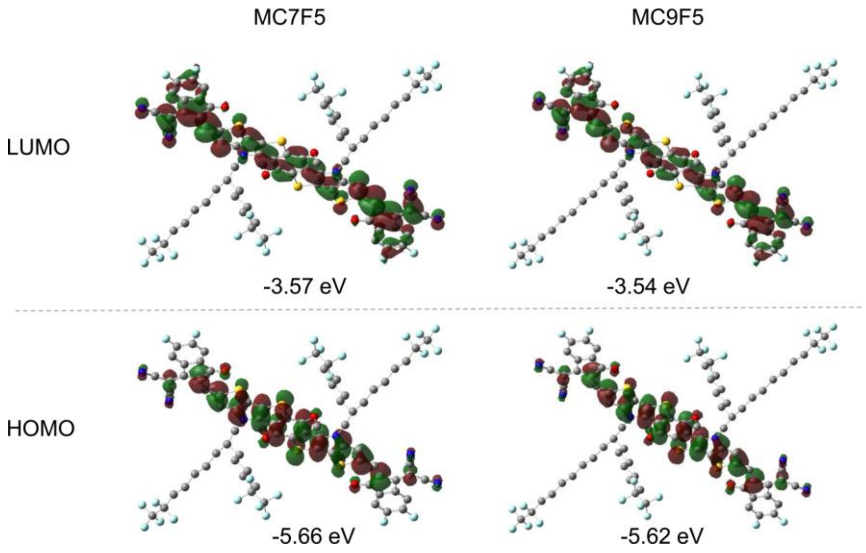

Figure S2. Calculated HOMO and LUMO energy levels of MC7F5 and MC9F5.

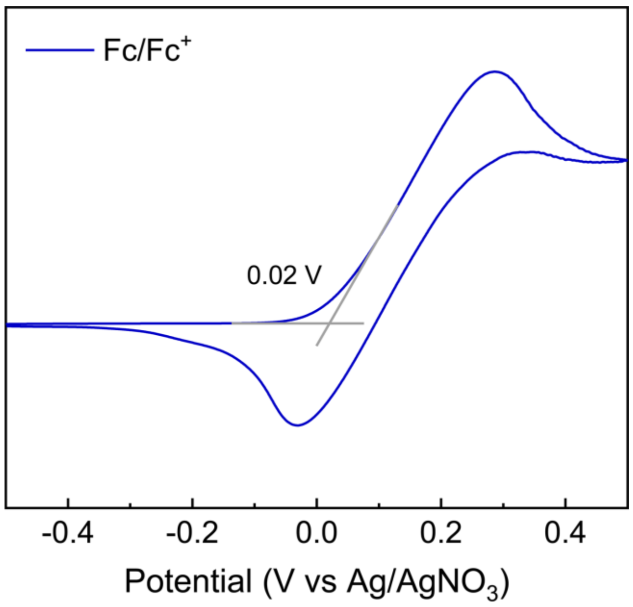

Figure S3. The cyclic voltammogram of Fc/Fc^+ couple.

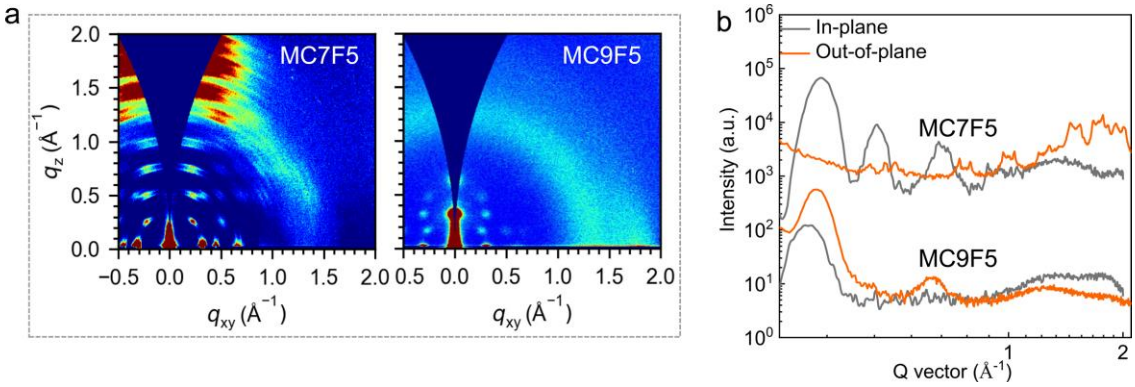

Figure S4. (a) Two-dimensional GIWAXS patterns and (b) in-plane and out-of-plane line-cuts of the neat MC7F5 and MC9F5 films.

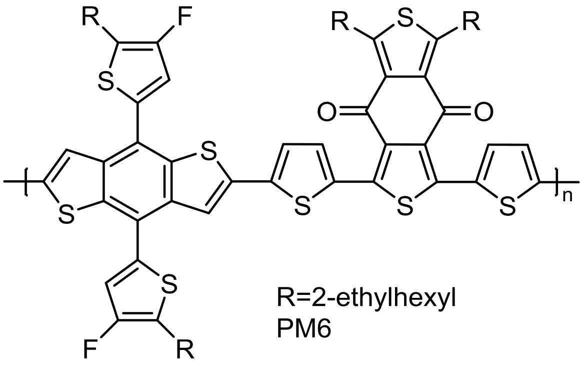

Figure S5. The molecular structure of PM6.

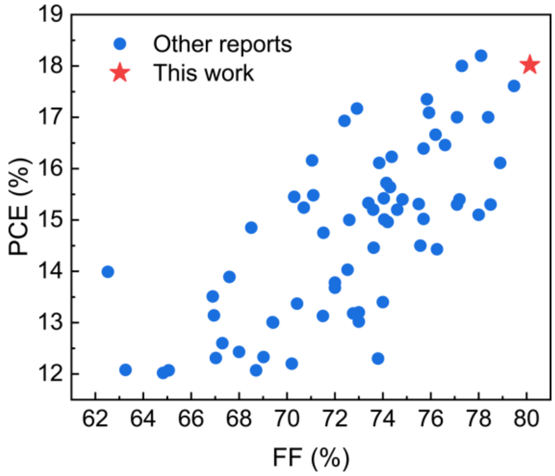

Figure S6. The PCE-FF data of OSCs based on linear ADA-type NFAs reported in recent years.

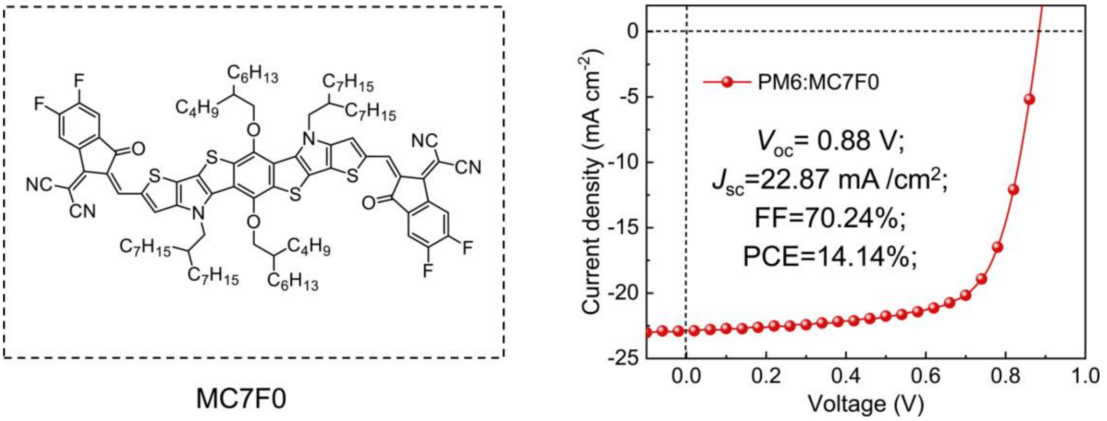

Figure S7. The molecular structure of MC7F0 (left) and J-V curve of the champion OSC based on PM6:MC7F0 (right), the corresponding photovoltaic parameters are also provided.

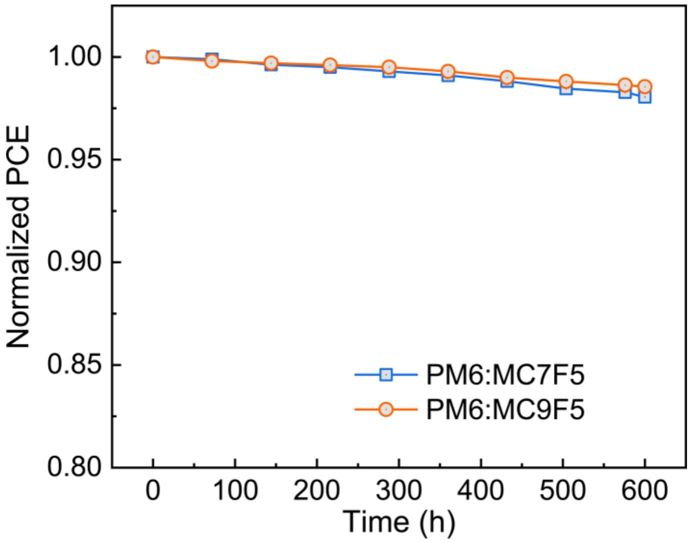

Figure S8. Normalized PCEs of the OSCs based on PM6:MC7F5 and PM6:MC9F5 after storage at room temperature in a N2-filled glovebox for different times.

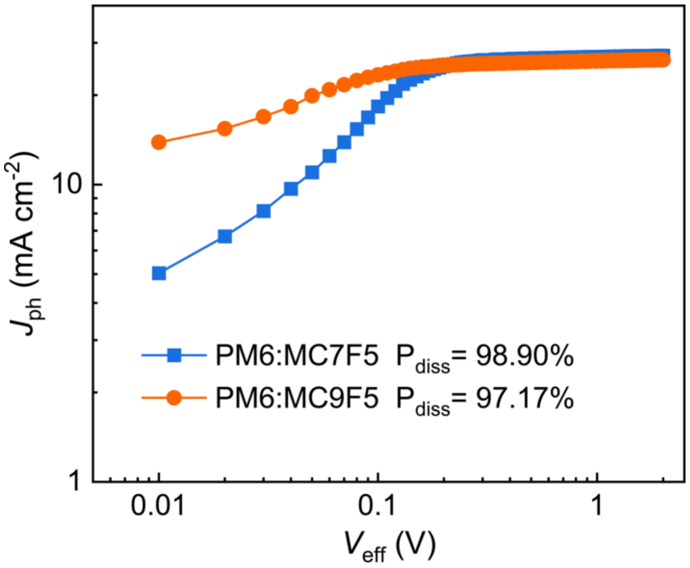

Figure S9. J_{ph}-V_{eff} characteristics of the optimized OSCs based on PM6:MC7F5 and PM6:MC9F5.

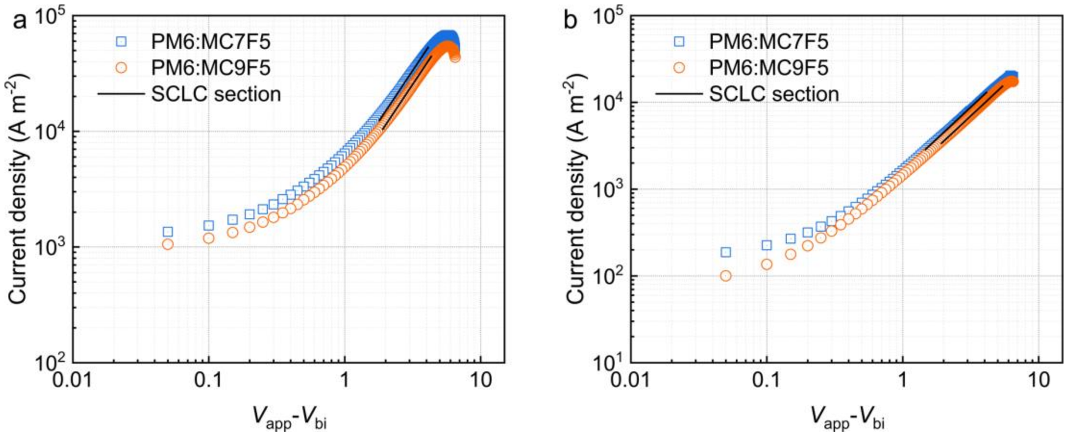

Figure S10. *J-V* curves of (a) hole-only and (b) electron-only devices based on PM6:MC7F5 and PM6:MC9F5 blend films under dark conditions.

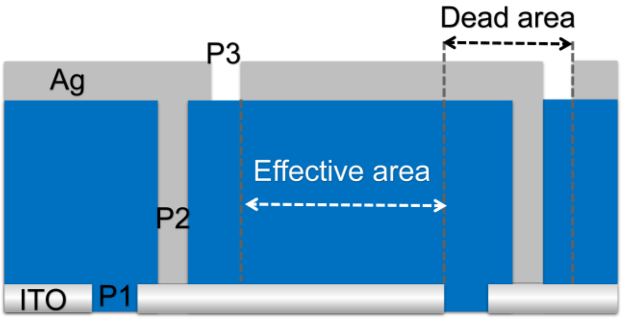

Figure S11. Schematic diagram of the structure of the module device with an

effective active layer comprising PEDOT:PSS/PM6:MC9F5/PDIN.

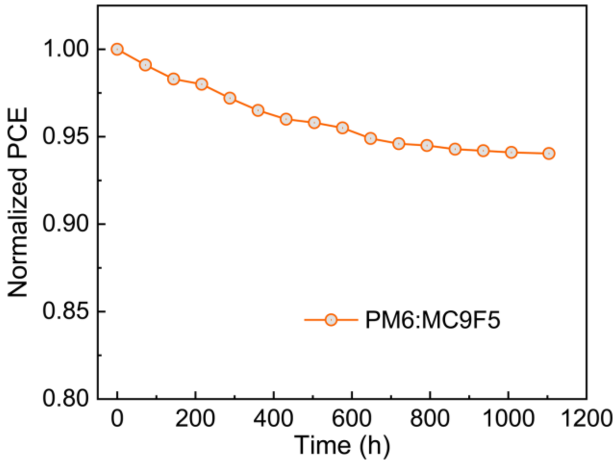

Figure S12. Normalized PCEs of the MC9F5-based module device after storage at room temperature in a N_2-filled glovebox for different times.

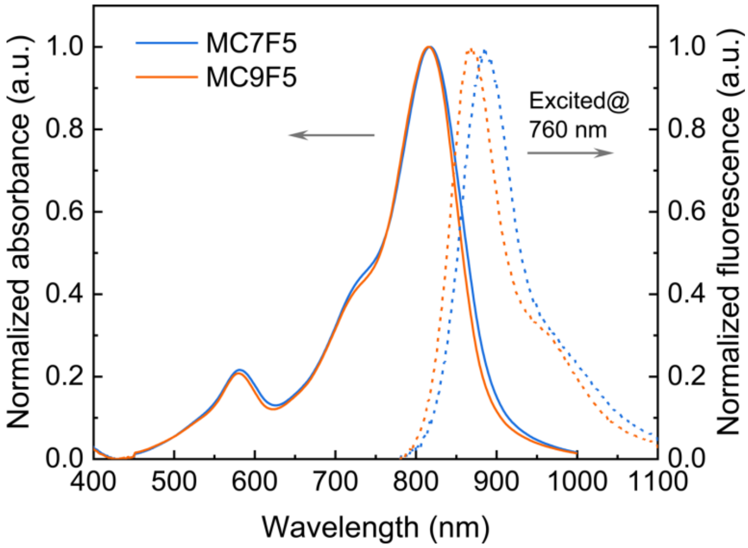

Figure S13. Normalized emission and absorption spectra of the neat MC7F5 and MC9F5 films.

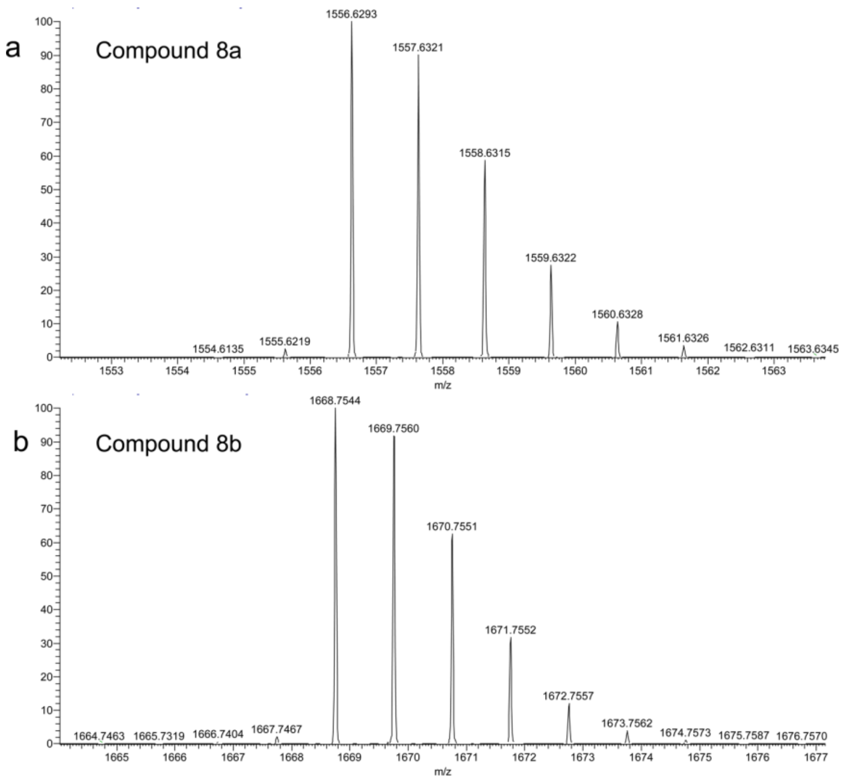

Figure S14. HRMS spectra of compounds (a) 8a and (b) 8b.

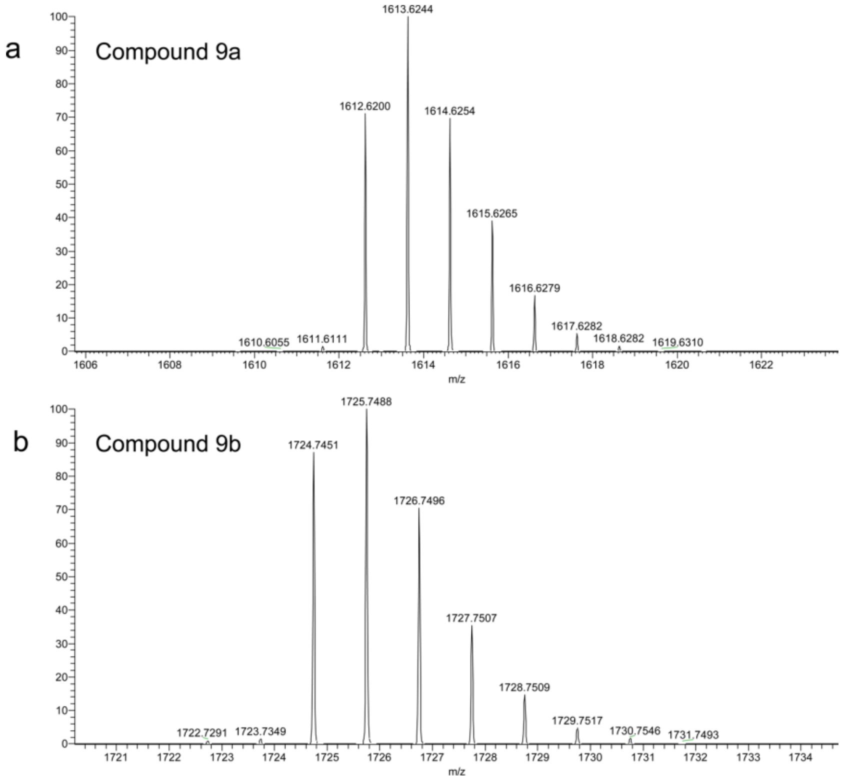

Figure S15. HRMS spectra of compounds (a) 9a and (b) 9b.

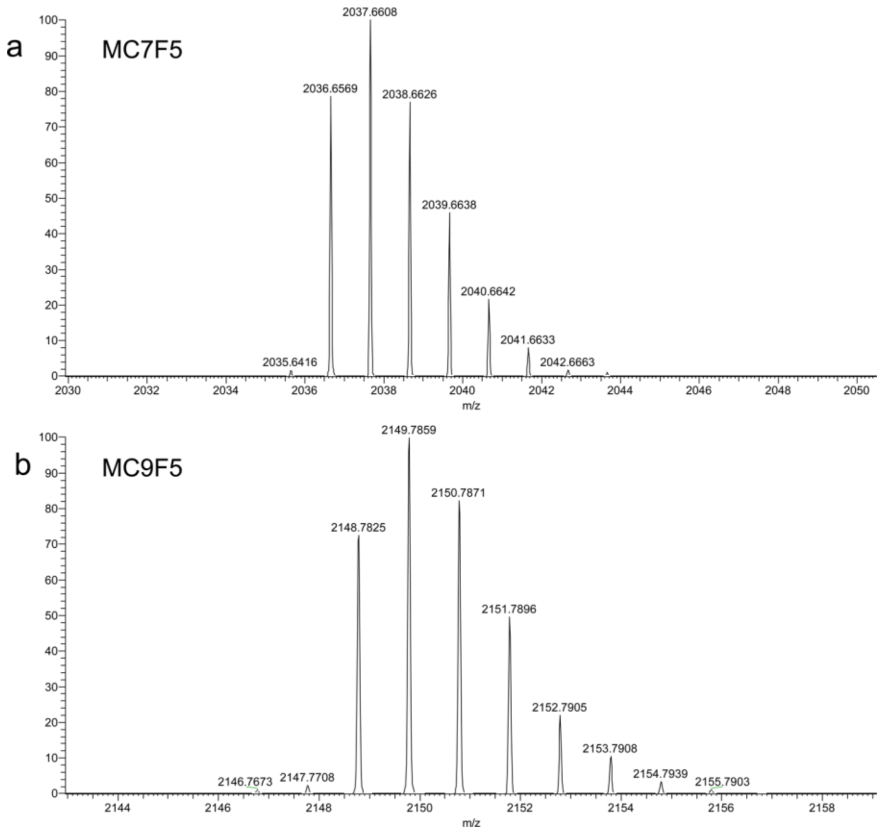

Figure S16. HRMS spectra of (a) MC7F5 and (b) MC9F5.

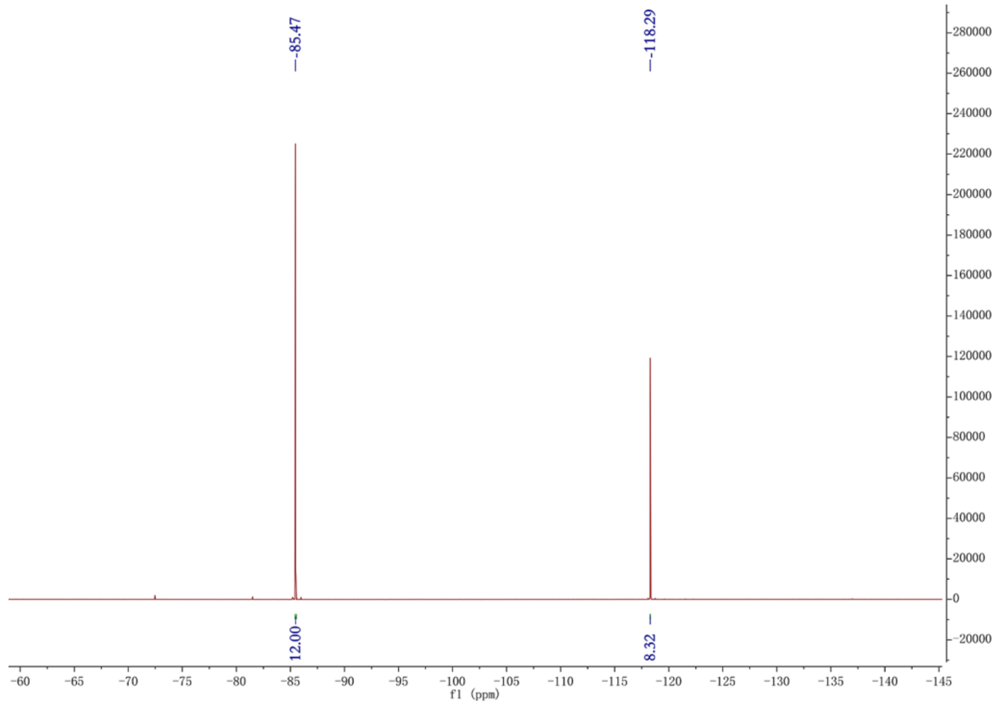

Figure S17. ^{19}F NMR spectrum of compound 8a.

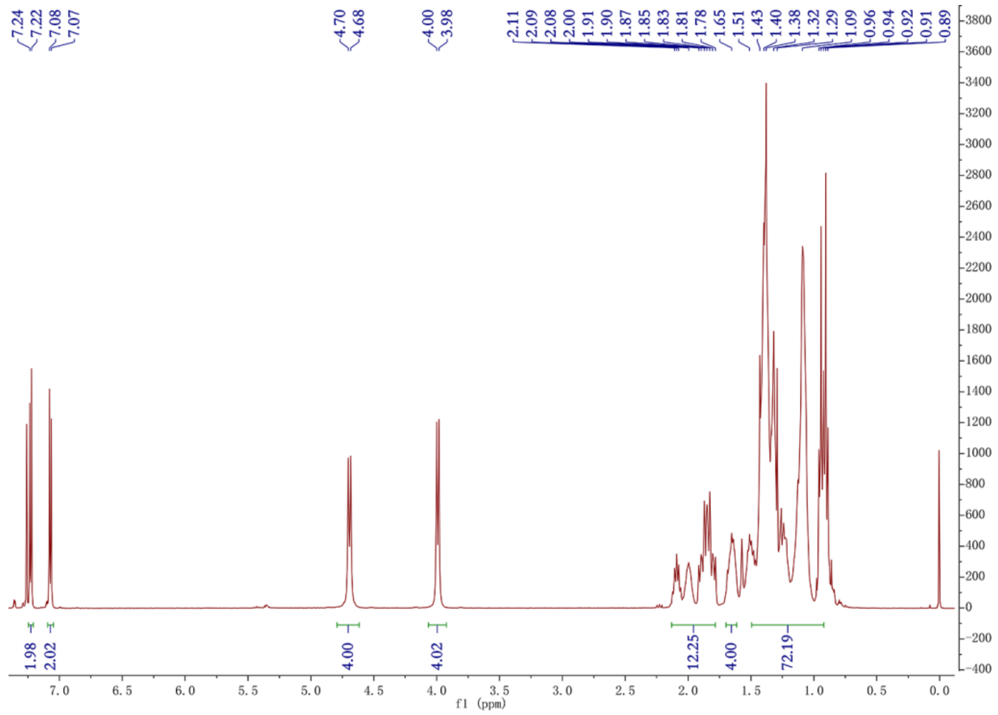

Figure S18. ^{1}H NMR spectrum of compound 8a.

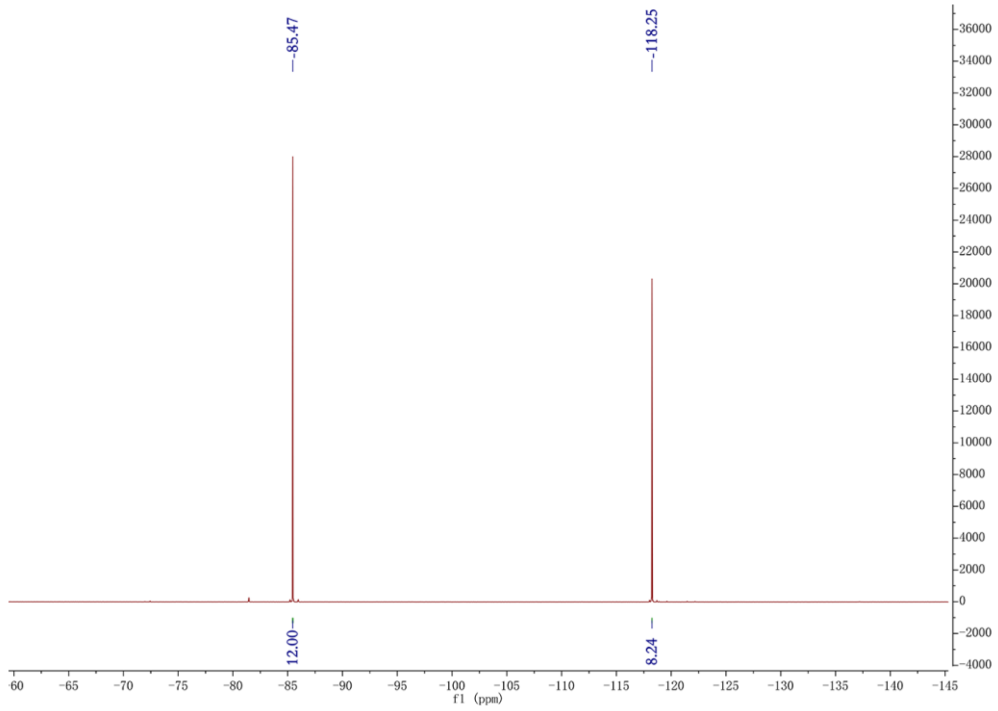

Figure S19. ^{19}F NMR spectrum of compound 8b.

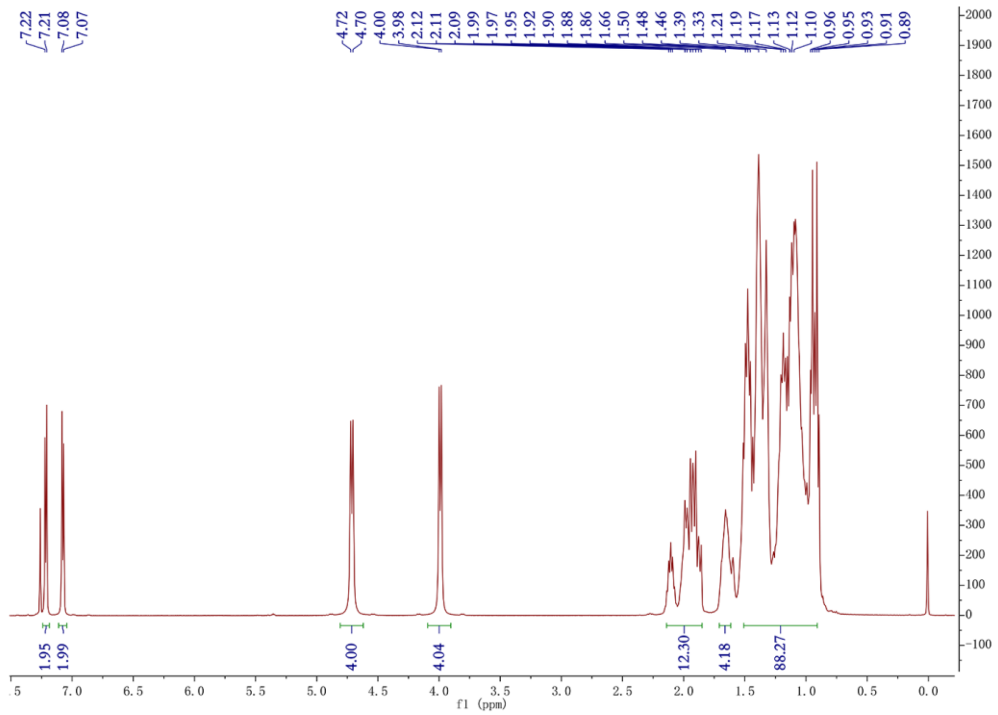

Figure S20. ^{1}H NMR spectrum of compound 8b.

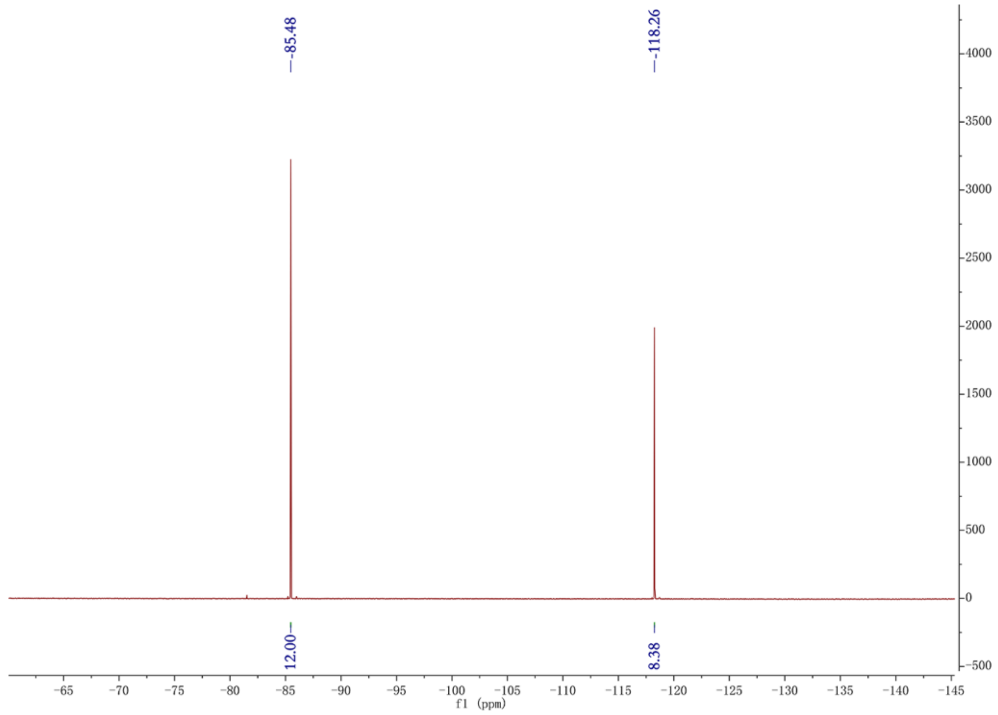

Figure S21. ^{19}F NMR spectrum of compound 9a.

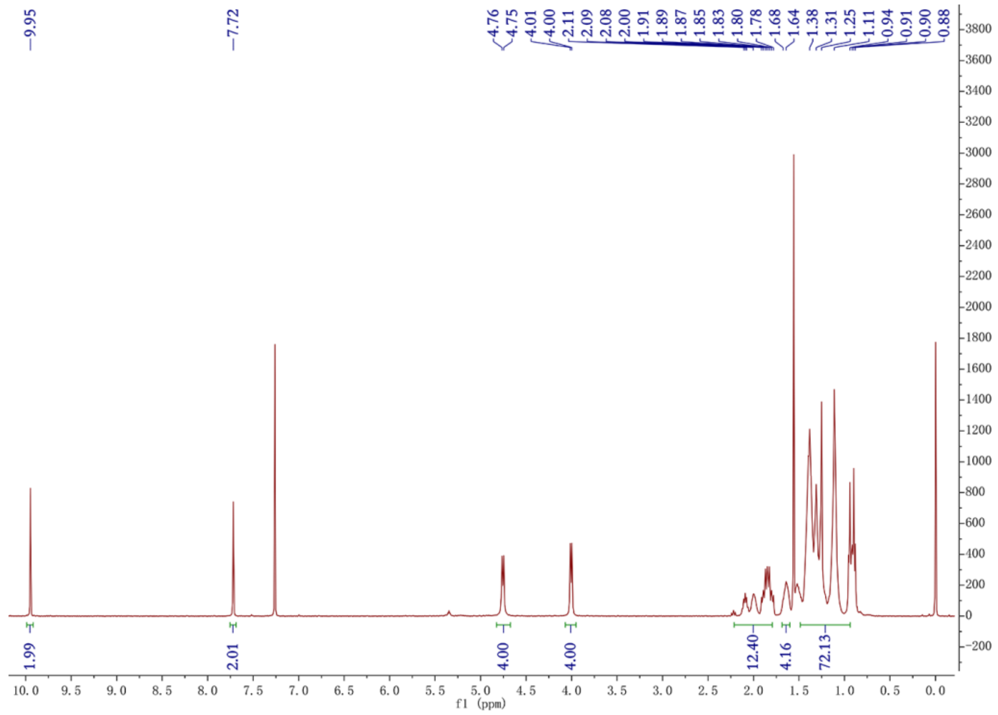

Figure S22. ^{1}H NMR spectrum of compound 9a.

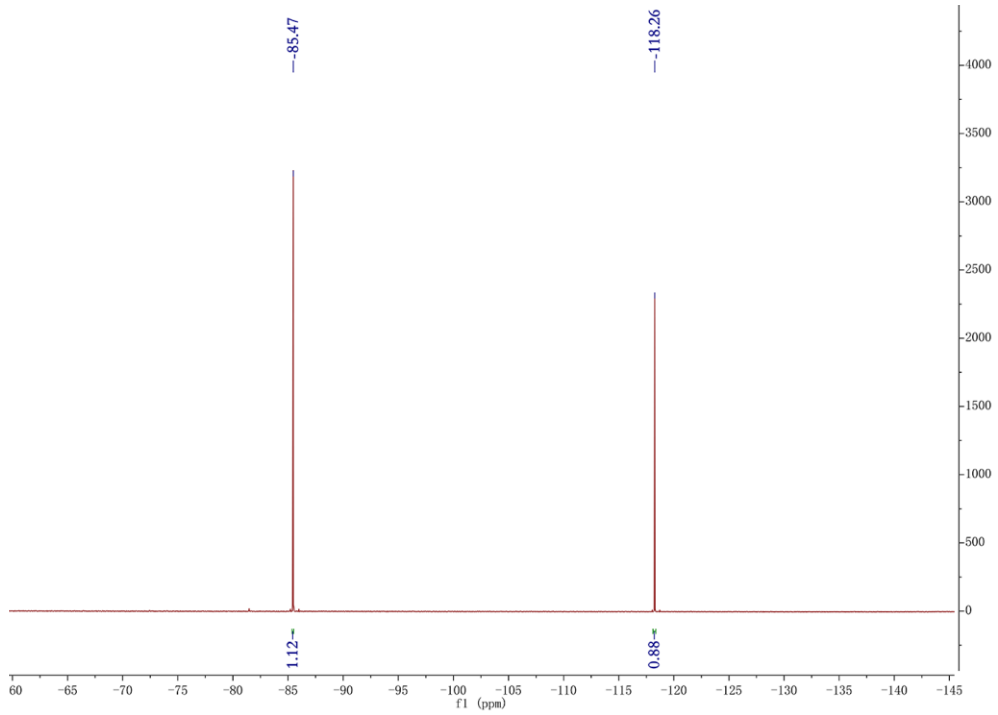

Figure S23. ^{19}F NMR spectrum of compound 9b.

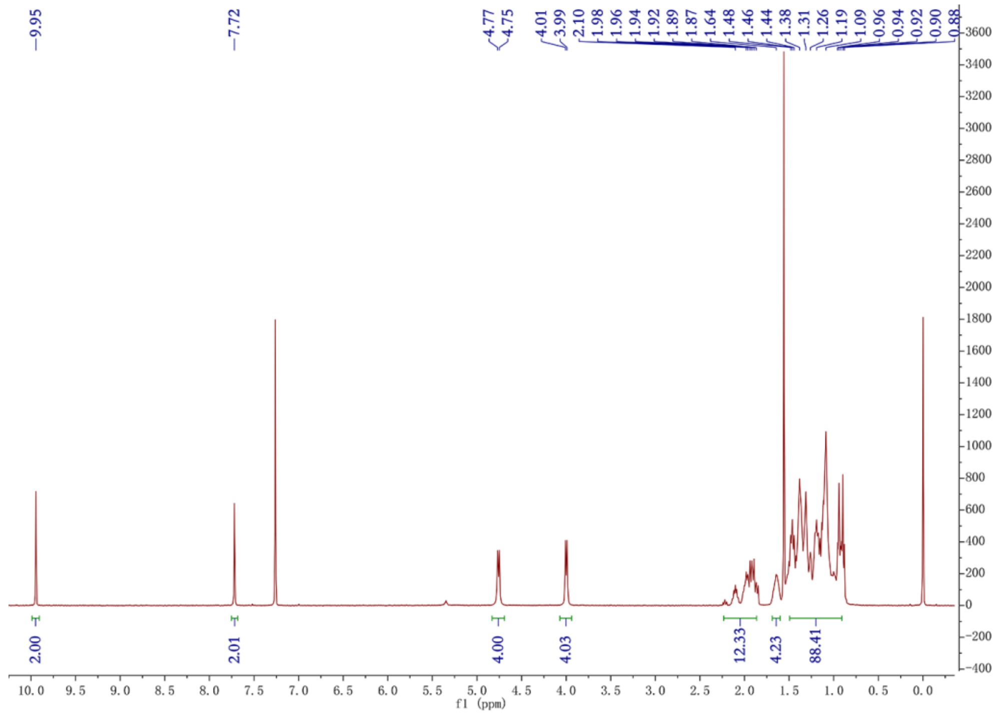

Figure S24. ^{1}H NMR spectrum of compound 9b.

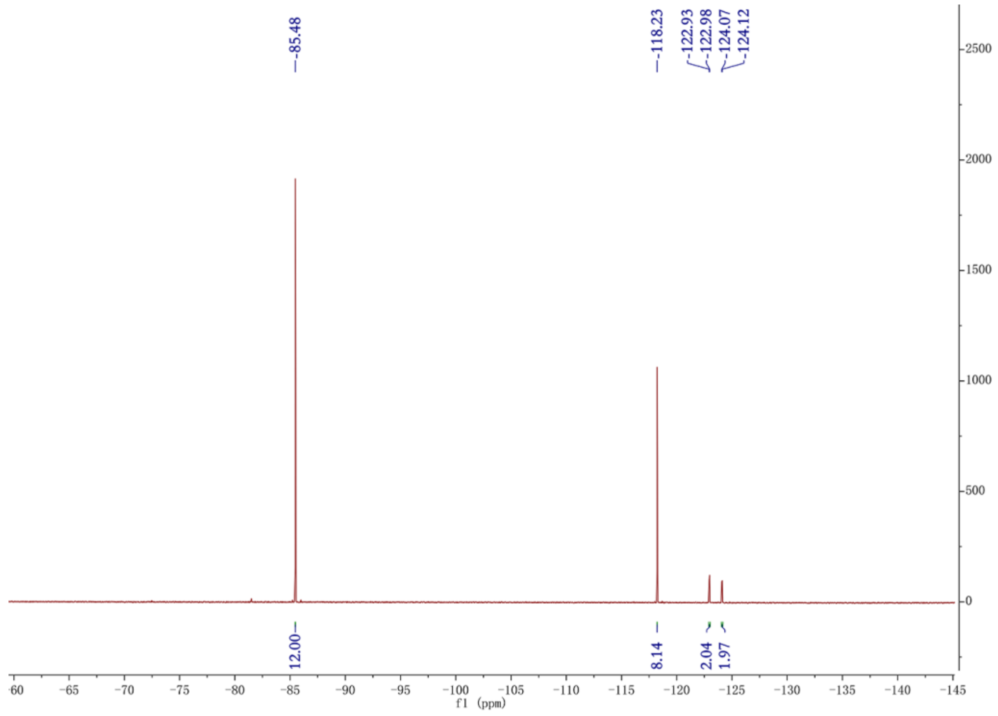

Figure S25. ^{19}F NMR spectrum of MC7F5.

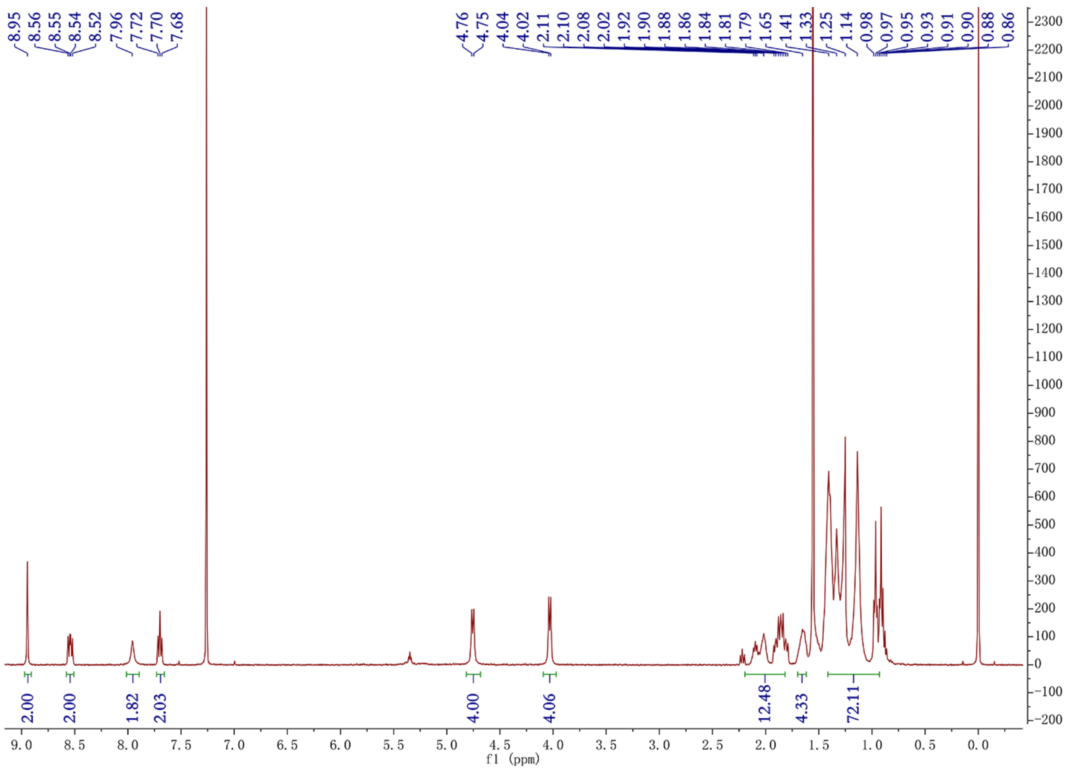

Figure S26. ^{1}H NMR spectrum of MC7F5.

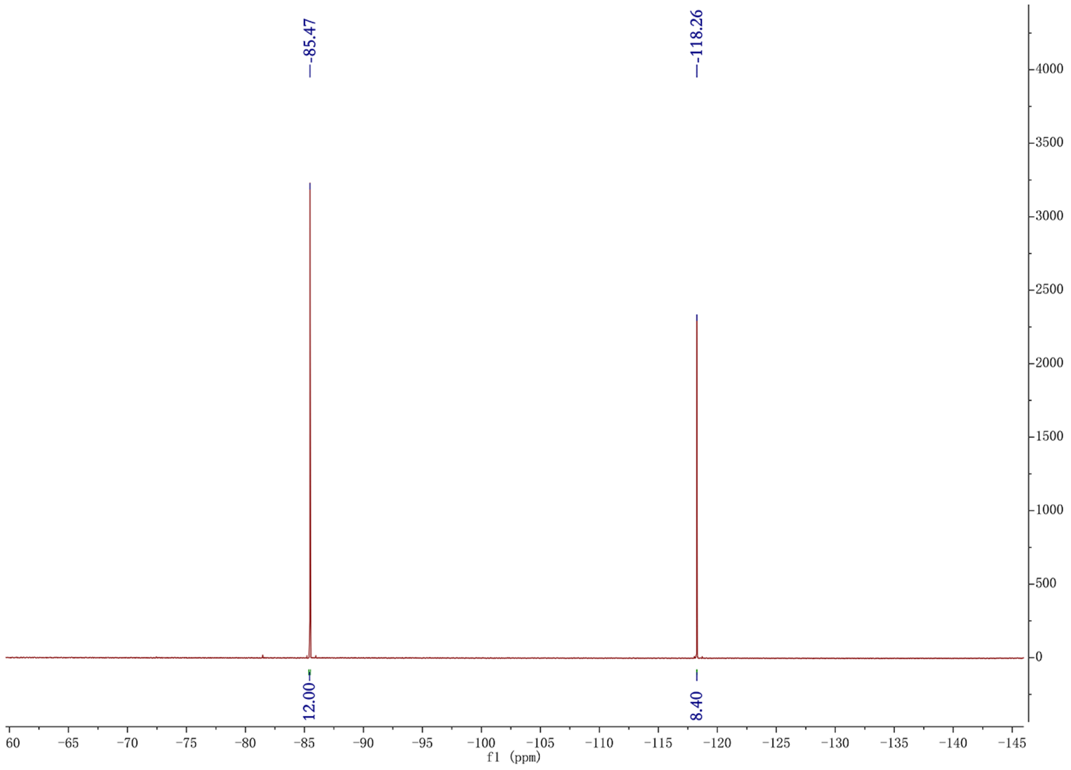

Figure S27. ^{19}F NMR spectrum of MC9F5.

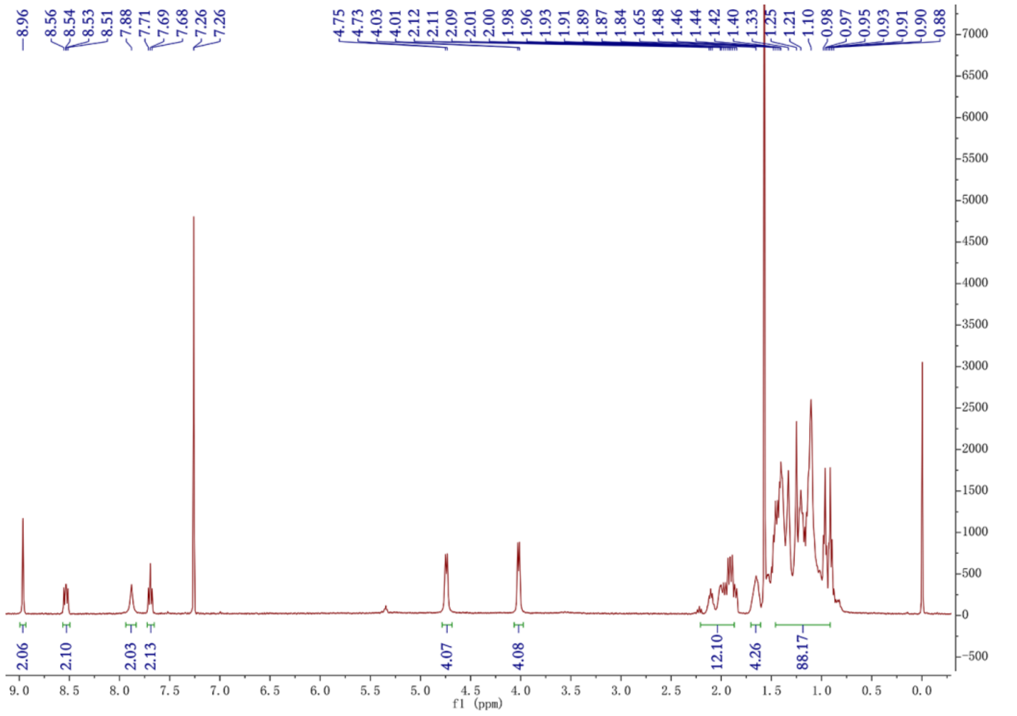

Figure S28. ^{1}H NMR spectrum of MC9F5.

Table S1. The summary of PCEs for the representative OSC modules.

Active layer	Active area (cm^2)	PCE (%)	Refs.
PM6:BTP-BO-4Cl[b]	18.73	14.79	*Adv. Mater.* **2022**, *34*, 2110569.
PM6:Y6:PCBM[b]	26.75	14.34	*J. Semicond.* **2022**, *43*, 100501.
PM6:T8[b]	7.50	14.62	*Nat. Energy.* **2022**, *7*, 1087.
PM6:Qx-1[b]	30.00	12.20	*Adv. Mater.* **2023**, *35*, 2209030.
PM6:CH7[b]	25.20	14.42	*Sol. RRL.* **2023**, *7*, 2300029.
PBQx-TF:eC9-2Cl[b]	23.60	15.10	*Adv. Mater.* **2023**, *35*, 2301583.
PM6:L8-BO[b]	18.73	15.20	*Adv. Mater.* **2023**, *35*, 2303729.
PM6:PBQx-TCl:PY-IT[b]	19.30	16.26	*Adv. Mater.* **2024,** *36*, 2308061.
PM6:G-Trimer[b]	46.20	13.25	*Joule.* **2023**, *7*, 2386.
PM6:NAP-TT-SiBTZ:Y7[b]	55.00	13.88	*Adv. Energy Mater.* **2023**, *13*, 2302538.
PM6:BTP-eC9[b]	16.94	14.58	*Adv. Mater.* **2024**, *36*, 2313098.
PM6:D18:L8-BO[b]	15.64	16.03	*Energy Environ. Sci.* **2024**, *17*, 2935.
PM6:L8-BO[b]	11.30	16.10	*Cell Rep. Phys. Sci.* **2024**, *5*, 101883.
PM6:L8-BO[b]	11.70	16.02	*ACS Nano* **2024**, *18*, 28026.
PM6:MC9F5[a]	11.09	15.66	**This work**

[a] ADA-type nonfullerene acceptor; [b] ADA'DA-type nonfullerene acceptor (Y-series acceptor).

Table S2. Optical and electrochemical properties of MC7F5 and MC9F5.

Acceptor	$\varepsilon_{max}^{solution}$ (M^{-1} cm^{-1})	$\lambda_{max}^{solution}$ (nm)[a]	ε_{max}^{film} (cm^{-1})	λ_{max}^{film} (nm)	E_g^{opt} (eV)[a]	HOMO (eV)[b]	LUMO (eV)[b]
MC7F5	1.98×10^5	731	2.42×10^5	818	1.36	-5.58	-4.07
MC9F5	2.13×10^5	735	2.82×10^5	816	1.38	-5.52	-4.02

[a]Optical bandgap determined by λ_{onset} in thin films; [b]Measured by cyclic voltammetry.

Table S3. The parameters of diffraction peaks from GIWAXS line-cuts of out-of-plane and in-plane profiles.

Samples	π-π stacking		Lamellar stacking	
	d-spacing (Å)	CL (Å) (FWHM)[c]	*d*-spacing (Å)	CL (Å) (FWHM)[c]
MC7F5[a]	/	/	19.87	120.32 (0.047 $Å^{-1}$)
MC9F5[b]	3.49	14.46 (0.391 $Å^{-1}$)	20.00	102.82 (0.055 $Å^{-1}$)
PM6:MC7F5[a]	3.59	18.85 (0.364 $Å^{-1}$)	21.58	102.76 (0.055 $Å^{-1}$)
PM6:MC9F5[a]	3.63	16.44 (0.301 $Å^{-1}$)	21.22	88.20 (0.064 $Å^{-1}$)

[a]The (010) diffraction peak along the q_z axis, (100) diffraction peak along the q_{xy} axis; [b]The (010) diffraction peak along the q_{xy} axis, (100) diffraction peak along the q_z axis; [c]Coherent length (CL)

estimated from the Scherrer equation (CL = $2\pi K$/FWHM, K = 0.9).

Table S4. Photovoltaic properties of PM6:MC9F5-based OSCs with different annealing temperatures.[a]

Temperature (^{o}C)	V_{oc} (V)	J_{sc} (mA/cm^2)	FF (%)	PCE[b] (%)
Room temperature	0.893	25.00	77.49	17.29 (17.19±0.11)
60	0.879	25.41	78.83	17.52 (17.42±0.15)
80	0.873	25.80	80.06	18.02 (17.91±0.08)
100	0.867	25.80	79.38	17.76 (17.44±0.18)

[a]MC9F5 was dissolved in chlorobenzene with 0.5 vol% CN as the additive, and the annealing time was set at 5 min; [b]Average PCEs with standard deviations in the parentheses are based on 8 devices.

Table S5. Photovoltaic properties of PM6:MC9F5-based OSCs with different amounts of CN additive.[a]

Additive	Content	V_{oc} (V)	J_{sc} (mA/cm^2)	FF (%)	PCE[b] (%)
	0	0.885	24.78	77.44	16.98(16.73±0.16)
CN (vol%)	0.5	0.873	25.80	80.06	18.02 (17.91±0.08)
	1	0.870	25.57	79.18	17.61 (17.32±0.21)
TCB (mg/ml)	15	0.861	25.29	78.30	17.06 (16.80±0.16)

[a]The acceptor was dissolved in chlorobenzene with different amounts of CN, and the active layers were annealed at 80 ^{o}C for 5 min. [b]The average PCEs with standard deviations in the parentheses are based on 8 devices.

Table S6. Statistical sheet of PCE *versus* FF of the reported ADA-type NFA-based organic solar cells with PCEs > 12%.

Active layer	V_{oc} (V)	PCE (%)	FF (%)	Ref.
PM6:MC9F5	0.88	18.02	80.1	**This work**
PM6:MC7F5	0.83	17.20	78.9	**This work**
PBDB-T:SN6C9-4F	0.74	12.07	68.7	*Chin. J. Chem.* **2025**, *43*, 13.
PBQx-TF:TBB	0.90	16.2	74.0	*CCS Chem.* **2024**, doi: 10.31635/ccschem.024.202303631.
D18:C-F	0.86	15.4	77.2	*ACS Mater. Lett.* **2024**, *6*, 2100.

D18:S-F	0.92	17.0	77.1	
PM6:PTBTT-4F	0.88	14.50	75.6	*Small* **2024**, *20*, 2305529.
PM6:PTBTT-4Cl	0.85	14.03	72.5	
PM6:TPBTT-4F	0.89	15.72	74.2	
PM6:TPBTT-4Cl	0.86	14.85	68.5	
PM6:DMT-HF	0.92	17.17	72.9	*Angew. Chem. Int. Ed.* **2024**, doi: 10.1002/anie.202411155.
PM6:M36:PW-Se	0.89	18.00	77.3	*Adv. Mater.* **2024**, *36*, 2314169.
PM6:M36	0.91	18.2	78.1	*Adv. Energy Mater.* **2024**, *14*, 2401816.
PM1:MC7F3	0.86	17.61	79.5	*Chem* **2024**, *10*, 3131.
D18:IMC8-4Cl	0.97	13.99	62.5	*ACS Mater. Lett.* **2024**, *6*, 2100.
D18-B:BDOTP‐1	0.94	16.93	72.4	*Carbon Energy* **2023**, *5*, e250.
D18-B:BDOTP‐2	0.98	15.48	71.1	
D18:ZITI-N-6F	0.88	16.11	73.8	*Fundam. Res.* **2023**. doi:10.1016/j.fmre.2023.03.010.
D18:ZITI-N-4F/ D18:ZITI-N-6F	0.91	17.09	75.9	
D18:ZITI-N-8F	0.84	15.20	73.6	
PBDB-T:MD1T	0.76	12.43	68.0	*Aggregate* **2023**, *4*, e322.
PM6:M36	0.91	17.02	78.4	*Nano Energy* **2023**, *107*, 108116.
PM6:MQ1-δ	0.91	12.08	63.3	*Chin. Chem. Lett.* **2023**, *34*, 108448.
PM6:ThPy6	0.87	16.11	78.9	*Adv. Funct. Mater.* **2022**, *32*, 2203200.
PM6:IDTP-4F	0.89	15.02	75.7	
PM6:TIT-2FIC	0.91	13.00	69.4	*Chem. Eng. J.* **2022**, *427*, 131674.
PM6:ThPy2	0.85	12.30	73.8	*Natl. Sci. Rev.* **2022**, *9*, nwac076.
PM6:ThPy3	0.83	15.30	77.1	
PBDB-T:PTBTP-4F	0.86	12.33	69.0	*Org. Electron.* **2022**, *103*, 106461.
PM6:2PIC	0.91	12.60	67.3	*Chin. J. Chem.* **2022**, *40*, 2861.
PM6:M14	0.89	16.46	76.6	*CCS Chem.* **2022**, *5*, 455.
PM6:M17	0.87	13.01	69.4	
PM6:MQ7-i	0.87	16.23	74.4	*J. Mater. Chem. A* **2022**, *10*, 23915.
J71:*cis*-MF	0.85	12.31	67.0	*Chem. Eng. J.* **2022**, *432*, 134393.
PM6:ML-2FM	0.88	15.33	73.4	*Solar RRL* **2022**, *6*, 2200119.
PM6:MC1	0.91	12.02	64.8	*Chem. Eng. J.* **2022**, *427*, 131022.
PM6:MS1	0.91	15.01	74.1	
PM6:M3	0.91	16.66	76.2	*Joule* **2021**, *5*, 197.
PM6:M6	0.89	15.45	70.3	*Adv. Funct. Mater.* **2021**, *31*, 2010436.
PM6:MQ5:M36	0.89	17.24	76.0	*ACS Appl. Mater. Interfaces*, **2021**, *13*, *57684*.
PM6:M13	0.88	13.14	67.0	*Chem. Eng. J.* **2021**, *418*, 129497.
PM6:MQ3	0.91	13.51	66.9	*Angew. Chem. Int. Ed.* **2021**, *60*, 19314.
PM6:MQ5	0.86	15.64	74.3	
PM6:MQ6	0.88	16.39	75.7	
PFBCPZ:IT4F	0.92	15.3	78.5	*Nano Energy* **2021**, *82*, 105679.
T1:DTTC-4Cl	0.94	14.43	76.3	*Solar RRL* **2020**, *4*, 2000357.
T1:DTSiC-4Cl	1.00	14.46	73.6	

PM6:DTTC-4F	0.95	13.89	67.6	
PM6:DTTC-4Cl	0.92	15.42	74.0	*J. Mater. Chem. A* **2020**, *8*, 1131.
PM6:DTC-4F	0.94	13.37	70.4	
PM7:4TCIC-4F	0.94	13.02	73.0	*Solar RRL* **2020**, *4*, 1900417.
PM6:IPTBO-4Cl	0.89	15.00	72.6	*J. Mater. Chem. A* **2020**, *8*, 5458.
PM6:IPT-4F	0.91	14.96	74.2	
PM7:IDTP-4F	0.90	15.20	74.6	*Adv. Funct. Mater.* **2020**, *30*, 2000383.
PM7:TPIC-4Cl	0.88	15.31	75.5	*J. Mater. Chem. A* **2020**, *8*, 5927.
PTB7-Th:P6IC	0.69	12.20	70.2	*ACS Appl. Mater. Interfaces* **2020**, *12*, 14029.
PM6:M4	0.88	14.75	71.5	*J. Mater. Chem. A* **2020**, *8*, 24543.
PM6:M34	0.91	15.24	70.7	*Angew. Chem. Int. Ed.* **2020**, *59*, 21627.
PM6:M36	0.90	16.00	72.1	*Natl. Sci. Rev.* **2020**, *7*, 1886.
J71:ZITI-N	0.88	13.68	72.0	*iScience* **2019**, *19*, 883.
J71:ZITI-C	0.85	13.18	72.7	
PM6:CZTT-4F	0.94	12.07	65.1	*J. Mater. Chem. A* **2019**, *7*, 21903.
T1:IT4F	0.90	15.1	78.0	*Adv. Mater.* **2019**, *31*, 1808356.
PBDB-T:IPIC-4Cl	0.81	13.40	74.0	*Adv. Funct. Mater.* **2019**, *29*, 1903269.
PBDB-T:INPIC-4F	0.85	13.13	71.5	*Adv. Mater.* **2018**, *30*, 1707150.
PBDB-T:SN6IC-4F	0.78	13.20	73.0	*Chem. Mater.* **2018**, *30*, 5429.

Table S7. Photovoltaic properties of PSCs based on PM1:MC9F5 and D18:MC9F5.

BHJ	V_{oc} (V)	J_{sc} (mA/cm^2)	FF (%)	PCE (%)[a]
D18:MC9F5	0.859	23.92	77.92	16.01 (15.82±0.20)
PM1:MC9F5	0.880	22.63	75.76	15.10 (14.86±0.15)

[a]In parentheses are average values based on 8 devices.

Table S8. Carrier mobilities of the PM6:MC7F5 and PM6:MC9F5 blend films.

Active layer	μ_h (×10^{-4} cm^2 V^{-1} s^{-1})	μ_e (×10^{-4} cm^2 V^{-1} s^{-1})	μ_h/μ_e
PM6:MC7F5	10.90 (9.56±0.47)	5.11 (4.86±0.17)	2.13
PM6:MC9F5	7.98 (7.69±0.14)	4.33 (4.18±0.13)	1.84

Table S9. Detailed E_{loss} parameters of the PM6:MC7F5- and PM6:MC9F5-based OSCs.

Devices	E_g^{PV} (eV)	E_{CT} (eV)	ΔE_{CT} (eV)	EQE_{EL} (%)	ΔE_1 (eV)	ΔE_2 (eV)	ΔE_3 (eV)

PM6:MC7F5	1.45	1.38	0.07	9.19×10^{-6}	0.263	0.086	0.290
PM6:MC9F5	1.47	1.44	0.03	3.17×10^{-5}	0.265	0.052	0.259

References

1. Ma Y, Cai D, Wan S *et al.* Ladder-Type Heteroheptacenes with Different Heterocycles for Nonfullerene Acceptors. *Angew Chem Int Ed* 2020; **59**: 21627-33.
2. Lin Y, Firdaus Y, Isikgor FH *et al.* Self-Assembled Monolayer Enables Hole Transport Layer-Free Organic Solar Cells with 18% Efficiency and Improved Operational Stability. *ACS Energy Lett* 2020; **5**: 2935-44.
3. Wang Y, Qian D, Cui Y *et al.* Optical Gaps of Organic Solar Cells as a Reference for Comparing Voltage Losses. *Adv Energy Mater* 2018; **8**: 1801352.
